# Lineage-specific positive selection on *ACE2* contributes to the genetic susceptibility of COVID-19

**DOI:** 10.1093/nsr/nwac118

**Published:** 2022-07-01

**Authors:** Yuwen Pan, Panhong Liu, Fang Wang, Peng Wu, Fanjun Cheng, Xin Jin, Shuhua Xu

**Affiliations:** Key Laboratory of Computational Biology, Shanghai Institute of Nutrition and Health, University of Chinese Academy of Sciences, Chinese Academy of Sciences, Shanghai 200031, China; College of Life Sciences, University of Chinese Academy of Sciences, Beijing 100049, China; The Third People's Hospital of Shenzhen, National Clinical Research Center for Infectious Disease, The Second Affiliated Hospital of Southern University of Science and Technology, Shenzhen 518112, China; Cancer Biology Research Center (Key Laboratory of the Ministry of Education), Tongji Hospital, Tongji Medical College, Huazhong University of Science and Technology, Wuhan 430030, China; Department of Gynecologic Oncology, Tongji Hospital, Tongji Medical College, Huazhong University of Science and Technology, Wuhan 430030, China; Department of Hematology, Union Hospital, Tongji Medical College, Huazhong University of Science and Technology, Wuhan 430022, China; School of Medicine, South China University of Technology, Guangzhou 510640, China; Department of Liver Surgery and Transplantation Liver Cancer Institute, Zhongshan Hospital, Fudan University, Shanghai 200032, China; State Key Laboratory of Genetic Engineering, Collaborative Innovation Center of Genetics and Development, Center for Evolutionary Biology, School of Life Sciences, Fudan University, Shanghai 200433, China; Human Phenome Institute, Zhangjiang Fudan International Innovation Center, and Ministry of Education Key Laboratory of Contemporary Anthropology, Fudan University, Shanghai 201203, China; School of Life Science and Technology, ShanghaiTech University, Shanghai 201210, China; Center for Excellence in Animal Evolution and Genetics, Chinese Academy of Sciences, Kunming 650223, China

**Keywords:** *ACE2*, COVID-19, genetic susceptibility, genetic diversity, natural selection, East Asia

## Abstract

The Angiotensin-Converting Enzyme-2 (*ACE2*) gene, located on Xp22.2, attracts a great deal of attention because the protein it encodes is believed to be the functional cellular receptor for the new coronavirus (SARS-CoV-2). However, recent studies are controversial, especially concerning the intrinsic link between *ACE2* diversity and COVID-19 susceptibility. Here, we conduct a population genetic study on *ACE2* in 6354 individuals representing 210 present-day populations and 5329 individuals of ancient or archaic groups. We dissected the genetic architecture of *ACE2* and identified two major haplogroups (hg) in East Asians, i.e. *ACE2*-hg1 (43%) and *ACE2*-hg2 (53%), while other populations harbor more diverse *ACE2*-hgs. Accordingly, there was a significant loss of *ACE2* common variations in East Asians in contrast to the X-chromosome-wide and genome-wide patterns. Notably, association analysis between *ACE2*-hgs and COVID-19 severity in 1229 Han Chinese individuals with various levels of COVID-19 severity showed a higher risk of *ACE2*-hg1 (odds ratio = 1.56, *P* < 0.01) and a lower risk of *ACE2*-hg2 (odds ratio = 0.65, *P* < 0.01). Interestingly, *ACE2*-hg1 is in strong linkage disequilibrium with rs1849863-C, which is an assumed risk factor of elevated plasma ACE2 level and is related to a higher risk of COVID-19 severity, hospitalization and infection. Strikingly, remarkable signatures of positive selection were detected, especially on *ACE2*-hg2, and were traced back to 100 000 years ago (but rose to a strong level during the Bronze Age, 5000∼3000 years ago, in East Asians). The selection pressures could have stemmed from multiple sources, but pre-COVID-19 viral epidemics and pandemics might have been potential driving forces, which consequently contributed to the genetic susceptibility to COVID-19 within and between populations.

## INTRODUCTION

Severe acute respiratory syndrome coronavirus 2 (SARS-CoV-2) in humans has resulted in the outbreak of coronavirus disease 2019 (COVID-19) and has already caused millions of deaths and hundreds of millions of confirmed cases around the world. Angiotensin-converting enzyme 2, the protein encoded by the *ACE2* gene (Xp22.2), was identified as the functional receptor for SARS-CoV-2 as well as the earlier SARS-CoV [1–4].

ACE2 is widely expressed in various organs, which provides possible routes of cell entry for SARS-CoV-2. Higher ACE2 expression was observed in organs other than the lung, such as the intestine, heart and kidney [5]. Many studies have been focusing on the genetic expression profiles of *ACE2* to predict COVID-19 susceptibility across worldwide populations, but these results remain controversial because a different relative abundance of ACE2 was inferred across populations [6–8].

Genetic variations of *ACE2* might account for COVID-19 susceptibility but the diversity of *ACE2* has yet to be fully understood. Some recent studies exclusively focused on searching for risk variants in *ACE2*. Nonetheless, the frequencies of loss-of-function (LoF)/missense variants are extremely low at *ACE2* [9,10]. In particular, none of the population-based genome-wide association studies showed a significant signature of *ACE2*-linked variants within or between populations [11–15]. A recent study also suggested a population-specific response to SARS-CoV-2 as East Asian populations have higher frequencies of the expression quantitative trait loci (eQTL) variants associated with higher *ACE2* expression [9]. Another study of the *ACE2* gene suggested that the susceptibility of South Asians is more similar to that of East Asians rather than West Eurasians, according to the haplotype similarity [16].

There are good reasons to believe that the genetic susceptibility to COVID-19 linked to *ACE2* was driven by some historical evolutionary events rather than being recently established due to a new coronavirus. ACE2 is also a primary modulator of the renin-angiotensin system (RAS) and plays a crucial role in regulating blood flow, pressure and fluid homeostasis. It has a beneficial role in many diseases such as hypertension, diabetes and cardiovascular disease, where its expression is decreased [17]. *ACE2* was shown to be associated with cardiovascular diseases, especially hypertension in rat models of high blood pressure [18]. Despite the potential importance of the *ACE2* gene in the cardiovascular system, controversial results were reported from association studies [19–23], likely due to differences in various factors such as subtypes of diseases, study design, population ethnicities and sample size.

Substantial disparities among different ethnic groups in COVID-19 outcomes have been reported by recent studies [24]. Therefore, in this study, we attempted to dissect the genetic architectures of the *ACE2* gene in worldwide populations, and systematically investigated the genetic diversity, haplotype structure and natural selection of *ACE2*. The purpose of this study is to identify the genetic determinants at *ACE2* associated with genetic susceptibility to COVID-19 and to elucidate the evolutionary mechanism of *ACE2* diversity within and between populations. In particular, we propose that some ancient natural selection on *ACE2* likely contributed to lower genetic susceptibility to COVID-19 in East Asia (EAS).

## RESULTS

### Dissecting the genetic structures of *ACE2*

**Figure 1.**
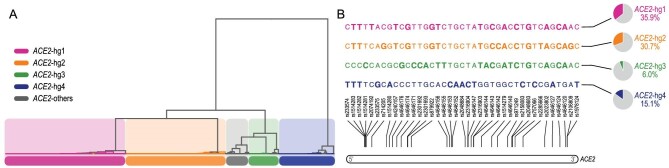
Dissecting the haplotype structures of *ACE2*. (A) Hierarchical clustering of *ACE2* sequences based on the identical-by-state distance matrix, where the branch lengths are not meaningful. (B) Haplotype structures of *ACE2* haplogroups formed by haplogroup informative markers (HIMs). Alleles in bold indicate the derived state (Methods).

The majority (∼88%) of *ACE2* sequences in worldwide populations can be clustered into four haplogroups, i.e. *ACE2*-hg1 (36%), *ACE2*-hg2 (31%), *ACE2*-hg3 (6%) and *ACE2*-hg4 (15%), as revealed by hierarchical clustering based on the identical-by-state (IBS) distance matrix (Methods, Fig. 1A) and confirmed with principal component analysis (PCA) (Fig. S1). Haplogroup informative markers (HIMs) were then determined based on the highly differentiated alleles across the four major haplogroups (Methods). An HIM was defined as a variant with one allele fixed in at least one of the haplogroups and the other allele fixed in the remaining haplogroups. A total of 39 HIMs of high quality and strong linkage were identified for further haplogroup classification (Fig. 1B, Supplementary Data, Table S1). Notably, *ACE2*-hg1 and *ACE2*-hg2 shared 87% of alleles at these HIMs.

**Figure 2.**
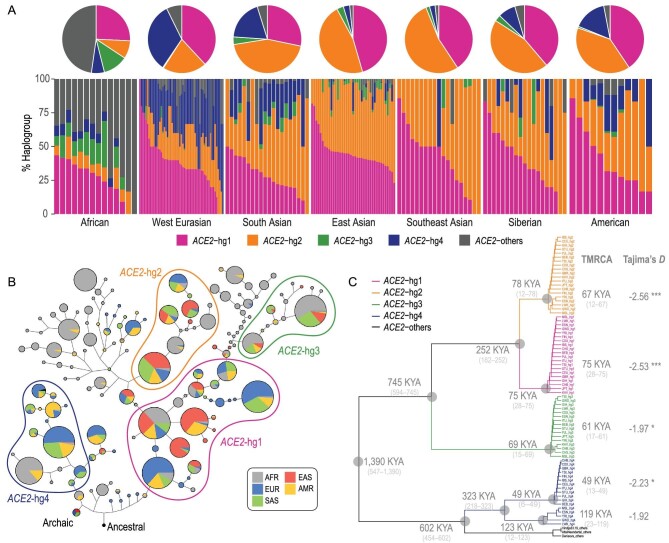
Haplogroup diversity of *ACE2*. (A) Haplogroup proportions of *ACE2* across worldwide populations. Each bar indicates the haplogroup makeups in a single population. Populations with >3 *ACE2* sequences are presented. Each pie indicates the mean haplogroup proportions averaged across all populations belonging to the same continental group. (B) Haplotype network of *ACE2* sequences. (C) Divergence tree of *ACE2* sequences across worldwide populations. Sequences from the same populations and of the same haplogroups are grouped and named after their population names and haplogroups, i.e. pop-hap. We excluded minor haplogroups in modern human populations from this analysis. Sequences of archaic hominids were used as outgroups. The tree topology was supported by 100 times bootstrapping replicates by random sampling of 20 sequences for each replicate from each pop-hap group. Within-pop-hap TMRCA and cross-pop-hap divergence time were averaged across all the replicates. The maximum TMRCA and divergence time in each clade are displayed at the corresponding node, and the ranges of dates are provided in brackets. Statistical significance: ^*^P < 0.05, ^**^P < 0.01, ^***^P < 0.001.

We further investigated the prevalence of *ACE2*-hgs across continental populations (Fig. 2A, Fig. S2). Notably, only two major haplogroups (43% *ACE2*-hg1 and 53% *ACE2*-hg2) were observed in EAS, with a third minor kind in low frequency (3% *ACE2*-hg3). In contrast, there were three major haplogroups (40% *ACE2*-hg1, 21% *ACE2*-hg2 and 31% *ACE2*-hg4) in West Eurasia (EUR), and a similar pattern in America (AMR). In South Asia (SAS), all of the four haplogroups are relatively common (24% *ACE2*-hg1, 47% *ACE2*-hg2, 8% *ACE2*-hg3 and 19% *ACE2*-hg4). As expected, haplogroup diversity is much larger in Africa (AFR); apart from the four common haplogroups (35% *ACE2*-hg1, 9% *ACE2*-hg2, 13% *ACE2*-hg3 and 8% *ACE2*-hg4), a few other kinds in relatively low frequencies were also observed, accounting for a total of 34% in frequency. Interestingly, the frequency of *ACE2*-hg2 is significantly correlated with longitude in EAS populations (*r* = 0.53, *P* < 7.35 × 10^–4^) (Fig. S3). We observed higher frequencies of *ACE2*-hg2 in populations located further towards the east; in particular, *ACE2*-hg2 is most prevalent in the She (77%) and Japanese (63%) (Table S2).

### Ancestral origins and evolution of *ACE2* haplogroups

We constructed the haplotype network (Methods) for the *ACE2* sequences (Fig. 2B). *ACE2*-hg4 is found in the highest frequency in EUR and in a lineage different from that of the other three haplogroups since the early split. It also seems that *ACE2*-hg4 has a relatively higher genetic affinity with the archaic *ACE2* sequences but is not inherited from archaic introgression based on the analysis with *ArchaicSeeker2.0* [25]. *ACE2*-hg1 is much more basal than *ACE2*-hg2 and *ACE2*-hg3. Both *ACE2*-hg2 and *ACE2*-hg3 are likely to be derived from *ACE2*-hg1. Some other minor haplogroups might also be derived from *ACE2*-hg1, which was mainly found in AFR populations.

We further estimated the time to the most recent common ancestor (TMRCA) and divergence time for the *ACE2* sequences (Methods). A phylogenic tree of *ACE2* sequences was constructed using hierarchical clustering based on the pairwise divergence time (Fig. 2C, Fig. S4). The common ancestor of the four haplogroups was traced back to as early as 1400 KYA. Consistent with the haplotype network, *ACE2*-hg4 was located in an isolated lineage. It diverged with the other lineages comprising *ACE2*-hg1, *ACE2*-hg2 and *ACE2*-hg3, followed by the split from archaic *ACE2* sequences at ∼600 KYA. Sub-lineages of *ACE2*-hg4 were formed in ∼300 KYA and found in both AFR and non-AFR populations. In contrast, *ACE2*-hg3 diverged earlier from the others at ∼750 KYA, but *ACE2*-hg1 and *ACE2*-hg2 diverged more recently at ∼250 KYA. The TMRCA estimated for each of the three haplogroups in non-African populations was as recent as <75 KYA, consistent with the Out-of-Africa (OOA) dispersal.

**Figure 3.**
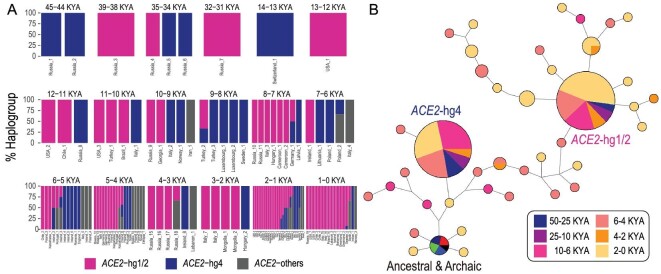
Haplotype make-up of *ACE2* in ancient humans. (A) Haplogroup proportions of *ACE2* sequences across the ancient groups. Ancient samples were grouped according to their dates and locations. (B) Haplotype network of ancient *ACE2* sequences.

Analyses of *ACE2* sequences in ancient samples indicated that *ACE2*-hg1/2 and *ACE2*-hg4 were already prevalent 50 000 years ago (KYA) in human populations (Fig. 3A), although *ACE2*-hg1 and *ACE2*-hg2 could not be distinguished in ancient DNA data [26] (Methods). Moreover, available sequences within the same haplogroups were identical in ancient samples dated from 50 KYA to those in present-day populations (Fig. 3B, Methods). Available archaic *ACE2* sequences are also almost identical with respect to the analyzed variants in modern human data.

Notably, excess rare variations were observed for all haplogroups in all populations, as also indicated by significantly negative Tajima's *D* statistics [27] (Fig. 2C, Methods). These results, especially the excess of rare variants and relatively small TMRCA, implied a recent selective sweep on the *ACE2* gene and the quick expansion of *ACE2*-hg1 and *ACE2*-hg2.

### Association between *ACE2* haplogroups and COVID-19 severity

We further employed the *ACE2* variations of Han Chinese COVID-19 patients [14,28,29] and examined the connections of *ACE2* haplogroups with COVID-19 severity. A total of 1229 samples (601 males and 628 females) were analyzed, with COVID-19 severity ranked as asymptomatic, mild, moderate, severe and critical. Detailed information is provided in the Supplementary Data (Fig. S5, Table S3). Notably, females tended to be mild, while males were likely to have higher severity (odds ratio (OR) = 1.40, *P* < 1.44 × 10^–3^). When individuals were grouped more broadly, i.e. asymptomatic, mild, moderate severities, and others, the pattern was retained (OR = 1.43, *P* < 2.07 × 10^–3^). These results are in agreement with previous findings [30], i.e. that there must be some protective mechanism in females. Only male samples were used in the following analyses to investigate the variations of severity across individuals and to eliminate the potential biases caused by the phasing error, unbalanced chromosome copies across genders, and tangles of different haplogroups in the same female individual (see Supplementary Data).

**Figure 4.**
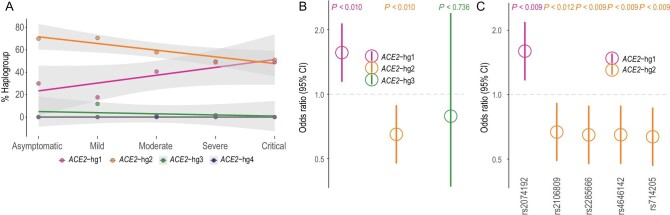
Association studies of COVID-19 severities with *ACE2* haplogroups. (A) Haplogroup proportions for patients of different severities. (B) Ordered logistic regression between COVID-19 severities and haplogroup type. OR = 1.56 for *ACE2*-hg1 and OR = 0.65 for *ACE2*-hg2. (C) Variant-based ordered logistic regression between COVID-19 severities and allele frequency of haplogroup-specific HIMs on *ACE2*-hg1 and *ACE2*-hg2. *P*-values labeled on the plot are BH-corrected. Age, comorbidity and the top five principal components were used as the covariates in the association analyses. OR = 1.60, 0.64, 0.65, 0.65 and 0.67 respectively for rs2074192, rs714205, rs4646142, rs2285666 and rs2106809.

We found that individuals with severe COVID-19 symptoms were more likely to be *ACE2*-hg1 carriers, and those with mild symptoms were more likely to be *ACE2*-hg2 carriers. Overall, there was a pronounced trend of increasing COVID-19 severity with an increased proportion of *ACE2*-hg1 and decreased proportion of *ACE2*-hg2 (Fig. 4A). When patients’ age, comorbidity (whether diagnosed or not) and ancestry (the top five principal components) were controlled, significant association (BH-corrected *P* < 0.01, OR = 1.56 for *ACE2*-hg1, OR = 0.65 for *ACE2*-hg2) was detected based on the ordered logistic regression between COVID-19 severities and haplogroup types (Fig. 4B). In addition, there was a significant association between COVID-19 severity and the haplogroup-specific HIMs of both *ACE2*-hg1 and *ACE2*-hg2 (Benjamini-Hochberg (BH)-corrected *P* < 0.01, OR = 1.60 for the *ACE2*-hg1-specific variant, OR = 0.63–0.67 for *ACE2*-hg2-specific variants) (Fig. 4C). Similar results were also obtained, with a logistic regression analysis, between COVID-19 severity and haplogroup type (Fig. S6), conditioning on age, comorbidity and ancestry. As supporting evidence, the association between COVID-19 severity and *ACE2*-hg1/*ACE2*-hg2 was also confirmed in European populations by analyzing data in the COVID19-hg database (released in October 2020) [31] (Table S4). In particular, the association was especially significant with the haplogroup-specific lead variants, such as rs2074192 (*ACE2*-hg1 specific, *β* = 0.038, *P* < 0.007), rs714205 (*ACE2*-hg2 specific, *β* = −0.063, *P* < 0.034), rs4646142 (*ACE2*-hg2 specific, *β* = −0.071, *P* < 0.013) and rs2285666 (*ACE2*-hg2 specific, *β* = −0.074, *P* < 0.010). Some clues were also detected regarding the association between *ACE2*-hg4 and COVID-19 severity in a significant variant, rs1514280 (*ACE2*-hg4 specific, *β* = −0.03, *P* < 0.019). In contrast, no significant association of COVID-19 severity was observed with *ACE2*-hg3.

The association between COVID-19 severity and *ACE2* haplogroup type is expected to be related to the expression level of ACE2 and the biological roles of *ACE2*-hg1 and *ACE2*-hg2. A recent study has reported the casual effect of elevated plasma ACE2 levels on COVID-19 severity, hospitalization and infection [32]. The odds ratio is ∼1.6, which is similar to the estimation in our study. The lead variant (rs1849863) for one of the most significant protein quantitative trait loci (pQTL) was ∼100 kilobases (kb) upstream to *ACE2*. The effect allele (C) of rs1849863 was able to upregulate the expression of ACE2 (*β* = 0.164), suggesting rs1849863-C as a risk factor. Analysis in our study revealed a considerably high linkage between the rs1849863-C variation and *ACE2*-hg1 (*β* ≈ 0.7–0.8, *P* < 10^–15^) (Fig. S7), while the connections between rs1849863-C and other haplogroups were much lower (*β* ≈ −0.2). Previous studies have reported the differentiated functions of alleles on *ACE2*-hg1 and *ACE2*-hg2 in the context of other diseases. For example, the T allele of rs2285666, carried by *ACE2*-hg2, is significantly associated with a lower risk of cardiovascular death in females of European ancestry [33]. In Han Chinese female subjects, alleles of both rs2074192 (T) and rs2106809 (A) that were carried by *ACE2*-hg1 were associated with reduced circulating angiotensin-(1-7) levels [34], which may partially account for the greater blood pressure response to changes in dietary sodium intake in the Chinese population [35] and also be responsible for the increased susceptibility to hypertension [36]. These findings suggest the differential risks of *ACE2*-hg1 and *ACE2*-hg2 to multiple human diseases.

### Significant loss of common variations at *ACE2* in EAS

Analysis of the genetic diversity of *ACE2* in worldwide populations (Methods) indicated the lowest genetic diversity in EAS populations, estimated by the pairwise difference (*θ_π_*), although there was a similar number of segregating sites (*θ_K_*) across populations in Eurasia (Fig. S8). These results indicated a loss of common or ancient variants at the *ACE2* gene in EAS populations. We further confirmed that the lower diversity of *ACE2* in EAS cannot be explained by X-chromosome-wide diversity with the confounding factors under control (Fig. S8, Methods), because an even lower genetic diversity of *ACE2* was observed compared with the chromosome-wide background, suggesting the loss of common variants in the *ACE2* gene driven by some local evolutionary force such as natural selection. Moreover, the relatively low cross-population variance of *ACE2* diversity also suggested that natural selection as a driving force of *ACE2* variations was prevalent in EAS.

**Figure 5.**
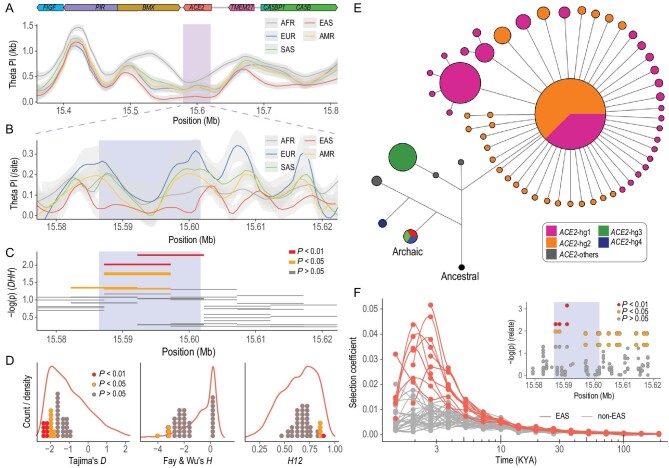
Natural selection signals of *ACE2*. (A) Nucleotide diversity for the neighboring region of *ACE2* estimated within sliding windows of 10 kb in length, advanced by 5 kb. (B) Site-based nucleotide diversity within the *ACE2* region. The selected region revealed by further analyses is highlighted. (C) Selection signals of *ACE2* in EAS indicated by *DHH* statistics (compound test combining Tajima's *D*, Fay and Wu's *H* and *H12* statistics). Each line denotes the *P*-value of *DHH* statistics estimated within a sliding window of 10 kb for a single population. (D) Distribution of Tajima's *D*, Fay and Wu's *H* and *H12* statistics of *ACE2* under the X-chromosome-wide background. Each dot denotes the statistical value of *ACE2* estimated within sliding windows of 10 kb in length by an increment of 5 kb for each population. The lines are the X-chromosome-wide distribution estimated in the same way. We grouped continental populations according to their similar profiles along the chromosome. (E) Haplotype network of the *ACE2* sequences in the region with significant *DHH* signals in EAS (chrX:15586447–15601720), where *ACE2*-hg1 and *ACE2*-hg2 shared the same HIMs. (F) Theoretical selection coefficients of *ACE2* variants (rs4240157, rs4646174 and rs879922) along with their history. The most significant signals of natural selection were identified in the three variants by Relate. Each curve indicates the changes in the selection coefficient of each variant in one single population. The selection coefficient was estimated based on the allele frequency changes inferred by Relate, assuming an additive model, without the consideration of genetic drift. The selected region (chrX:15586447–15601720) was highlighted.

To examine to what extent the selective sweep affected the flanking genes of *ACE2*, we scanned the region spanning ∼200 kb up and downstream of *ACE2*, encompassing *ACE2* and another six genes including *FIGF*, *PIR*, *BMX*, *TMEM27*, *CA5BP1* and *CA5B*. There was an obvious decrease in diversity at *ACE2* and *BMX* compared with other flanking genes, especially in EAS (Fig. 5A and B). *BMX* is in strong linkage with *ACE2* (Fig. S9) and encodes a non-receptor tyrosine kinase and participates in cardiac growth and endothelial signalling. Therefore, the two genes are highly relevant both physically and functionally, with natural selection affecting the diversity of both genes significantly. *TMEM27* is ∼30 kb upstream of *ACE2* and encodes a novel homolog of *ACE2*. However, no significant differentiation of allele or haplotype frequencies was observed among worldwide populations, and all the continental groups are of high nucleotide diversity at *TMEM27*. In addition, we collected 15 genes related to COVID-19 (COVID19Genes) (Table S5) and 64 genes related to immunity (ImmuneGenes) on the X chromosome (Methods). Compared with the X-chromosome-wide protein-coding genes, COVID19Genes and ImmuneGenes, *ACE2* also showed extremely low nucleotide diversity in EAS (Fig. S10).

### Positive selection on *ACE2*-hg2 in EAS

A compound test of natural selection based on *DHH* statistics combining Tajima's *D* [27], Fay and Wu's *H* [37,38] and *H12* [39] statistics was further applied to an analysis of *ACE2* sequence data (Methods). Previous studies have demonstrated the robustness and power of the joint test of both sites- and haplotype-frequency-based methods [38,40,41]. EAS-specific signals of positive selection were revealed by the *DHH* statistics (*P* < 0.006) (Fig. 5C, Fig. S11), consistent with the observation of the reduced diversity in *ACE2* in East Asian populations (Fig. 5A and B, Fig. S8).

The *DHH* signals were also supported by the three specific statistics (Fig. 5D, Figs S12 and S13). An excess of rare variants compared with common variants was indicated by the Tajima's *D* statistics (theoretical *P* < 0.001, Tajima's *D* < 0). The effect of demography was controlled based on the chromosome-wide data (empirical *P* < 0.009, Tajima's *D* < 0), with other confounding factors eliminated following the previous study [42] (Methods). The results of Fay and Wu's *H* statistics revealed an excess of variants with high derived allele frequency (empirical *P* < 0.03, Fay and Wu's *H* < 0). Moreover, haplotype homozygosity analysis with *H12* statistics suggested the dominance of the top two haplotypes in EAS (empirical *P* < 0.009).

Haplotypes of *ACE2* in the selected region (chrX:15586447–15601720) were clustered in a super branch in EAS (Fig. 5E). A great number of rare variants (i.e. singletons and doubletons) were observed on *ACE2*-hg1 and *ACE2*-hg2, implying their recent expansion. There were 44 (77.2%) branches observed on the node of the clustering center in EAS (Fig. 5E), while the numbers were much lower for other populations (≤30 branches (50%)) (Fig. S14). The EAS-specific excess of rare variants was also revealed by both Fu and Li's *D* statistics (empirical *P* < 0.005, Fu and Li's *D* < 0) and Fu and Li's *F* statistics (empirical *P* < 0.006, Fu and Li's *F* < 0) [43] (Figs S12 and S13), which were designed by comparing the intermediate/low frequency variants with singletons.

Despite the identical genetic background of *ACE2*-hg1 and *ACE2*-hg2 within the selected region, our analysis suggested *ACE2*-hg2 is most likely the main target of natural selection. A considerably higher frequency of *ACE2*-hg2 (53%) was observed in EAS compared to *ACE2*-hg1 (43%), though *ACE2*-hg1 was basal (Fig. 2B). In contrast, *ACE2*-hg1 was dominant in other populations, e.g. EUR (40% *ACE2*-hg1, 21% *ACE2*-hg2). The geographic distribution of *ACE2*-hg2 frequency in EAS (Fig. S3) also suggested a local adaptation on *ACE2*-hg2. In addition, haplogroup-specific Tajima's *D* statistics (empirical *P* < 0.005, Tajima's *D* < 0), Fu and Li's *D* statistics (empirical *P* < 0.002, Fu and Li's *D* < 0) and Fu and Li's *F* statistics (empirical *P* < 0.002, Fu and Li's *F* < 0) (Fig. S15) indicated a lineage-specific natural selection on *ACE2*-hg2. Correspondingly, we also observed excess singletons on *ACE2*-hg2 (Fig. S16), i.e. 64 singletons located on *ACE2*-hg2 compared with 34 on *ACE2*-hg1.

### Adaptive evolution of *ACE2* in EAS over the last 3000 years

We applied Relate analysis [44] to investigate historical evidence of selection on *ACE2*, following the estimate of population size with chromosome X data in the 1000 Genomes Project (Methods). Population sizes inferred from the X-chromosomal data (Fig. S17) and from autosomal data [44] were comparable. The results indicated the onset of selection in modern human populations over the last 100 000 years in both AFR and EAS populations. The footprints of natural selection could be found in *ACE2*-hg1, *ACE2*-hg2 and *ACE2*-hg3 (*P* < 0.01) (Fig. S18).

However, in EAS, strong signatures were observed in HIMs shared by both *ACE2*-hg1 and *ACE2*-hg2, i.e. rs4240157 (*P* < 0.005), rs4646174 (*P* < 0.005) and rs879922 (*P* < 0.001). The three HIMs were also located in the region with significant *DHH* signals (Fig. 5F) and were also confirmed to be the most likely causal variants in EAS with iSAFE (for `integrated selection of allele favored by evolution') analysis [45] (Fig. S19). The swiftest allele frequency increases occurred at ∼3–5 KYA in EAS (Fig. 5F, Fig. S18), roughly corresponding to the evolution of civilization in the Bronze Age. The selection coefficient reached 0.04 over the last 3000 years in EAS, assuming an additive model (Fig. 5F, Methods), or even greater under a dominant model (Fig. S18). The allele frequency changes inferred with Relate were also supported by the data of ancient samples across the Eurasian continent [26] (Fig. S20, Methods).

## DISCUSSION

In this study, we focused on the genetic diversity of the *ACE2* gene and analyzed one aspect of population genetics and molecular evolution. We were aware that, recently, many other genes have been reported to be potentially associated with COVID-19 susceptibility or severity. The heritability of severe COVID-19 susceptibility was estimated to be 6.5% based on genome-wide variations [11], suggesting a significant genetic contribution to COVID-19 susceptibility and severity. One reason why we focused on this particular gene, *ACE2*, was that we observed a connection between *ACE2* variants and COVID-19 severity in our data. *ACE2* is the key factor for the cell entry of SARS-CoV-2. Many studies have been trying to identify LoF/missense variants at *ACE2* with high allele frequency differences across worldwide populations [9,10], which might be an indicator of population-specific susceptibility. There were two strong assumptions behind the idea and design of these studies. One was that COVID-19 susceptibility/severity must be associated with some novel mutations, and the other was that human susceptibility to COVID-19 was just recently established. However, all the *ACE2* LoF/missense variants are of extremely low frequency among worldwide populations, preventing them from being potential genetic factors of COVID-19 susceptibility. Our analysis suggested that some regulatory variants at the intron region of *ACE2* are more likely to contribute to host susceptibility to COVID-19, and these variants are standing variants in high frequency rather than novel mutations in low frequency.

By dissecting the haplotype structure of *ACE2* across worldwide populations, we successfully connected the *ACE2* haplogroups with COVID-19 severity (Fig. 4B), which was also supported by the variant-based association analyses (Fig. 4C). These results highlight the genetic contributions of *ACE2* to COVID-19 severity. It is also a striking finding that *ACE2*-hg1 may function differently to *ACE2*-hg2, although they were recently diverged (∼250 KYA) and are in high sequence similarity (87% HIMs). In EAS populations, our data indicated high COVID-19 vulnerability of *ACE2*-hg1 carriers and low vulnerability of *ACE2*-hg2 carriers. The carriers of the two haplogroups, i.e. *ACE2*-hg1 and *ACE2*-hg2, are expected to experience different demography as *ACE2*-hg2 was under positive selection in EAS. Summary data from the COVID19-hg database also supported the same trend of the vulnerability associated with *ACE2*-hg1 and *ACE2*-hg2 in populations of EUR ancestry.

When the entangled multiple factors and models are simplified, the varying prevalence of *ACE2* haplogroups across global populations can be an indicator of population-specific susceptibility. EAS populations harbor both high-frequency *ACE2*-hg1 (43%) and *ACE2*-hg2 (53%), indicating differentiation of COVID-19 severities, i.e. the majority will have a lower risk of severe COVID-19, but another half will have a higher risk. In EUR populations, *ACE2*-hg1 is in similar frequency (40%) to EAS, but *ACE2*-hg2 is much lower (21%), which might mean a larger proportion of severe or critical patients. In SAS, *ACE2*-hg2 (47%) is more abundant than *ACE2*-hg1 (24%), meaning fewer severe or critical cases, relatively speaking. Despite this over-simplified model, the trends were supported by recent work that suggested SARS-CoV-2 may have a higher impact on the EAS-ancestry population than on the SAS-ancestry population [24]. The situation in African populations is more complex as the connection between diverse *ACE2*-hgs and COVID-19 severity has not been established yet. Despite the genetic contribution to COVID-19 severity being identified at *ACE2*, it would be noteworthy if other factors also played important roles, such as the influence of government policy and medical practice.

One of the most important findings of this study is that the genetic basis of susceptibility to COVID-19 via *ACE2* might have been established early in human evolutionary history. A recent study proposed that the common ancestor of primates was strongly resistant to SARS-CoV-2, and that New World monkeys were completely resistant, whereas apes and Old World monkeys, like most humans, are susceptible [46]. Several recent studies proposed that *ACE2* was under selection constraint in the human lineage [46,47]. Our data analysis also showed the extremely low allele frequencies of LoF/missense variants at *ACE2* among worldwide populations, and the relatively recent TMRCA of all major *ACE2* haplogroups. It is not unexpected, as ACE2 is a primary modulator of the RAS and plays a crucial role in regulating blood flow, pressure and fluid homeostasis. The functional conservation and importance of *ACE2* mean natural selection must have been common in driving its diversity. However, population differentiation of *ACE2* diversity also suggested ancestry-specific demography and local adaptation of *ACE2* variants or haplotypes.

Our analysis revealed that the frequency increases of *ACE2*-hg2 experienced an acceleration in the Bronze Age. It is not clear what kind of selection pressures have driven this process; human activities like civilization evolution could play an important role, and some ancient viral epidemics and pandemics also likely contributed to the patterns. We would point out that ancient purifying selection may couple with the later positive selection on *ACE2*, resulting in selection cancelation to some degree. Present-day data and currently available methods have limitations with regard to reconstructing the detailed process, and only captured the eventual consequence. Accordingly, we can detect the positive selection of *ACE2*-hg2 in EAS, which is the most likely scenario, but we do not reject the possibility of other selective sweeps—they might just be too weak to be detected. For example, balancing selection on both *ACE2*-hg1 and *ACE2*-hg2 could not be well supported by our analyses. Meanwhile, the genetic variation of *ACE2* in EAS was not likely to result from a negative selection, which cannot explain all the signals pointing to a positive selection. Suppose the negative selection has some considerable effects on *ACE2* in EAS populations; we would expect *ACE2*-hg3 to be the most likely target of negative selection due to its minor frequency in EAS (<5%). We would also expect the frequency of *ACE2*-hg3 to be different between males and females because males are haploid and are more likely to be influenced by deleterious mutations. It turned out that the data do not support the expectation, i.e. the frequency of *ACE2*-hg3 is very similar between males and females (∼2% in males and ∼3% in females). Similar frequencies were also observed between males and females for other haplogroups (Fig. S2). Other continental groups may have different scenarios. We have found that the pattern went in the opposite direction in EUR populations, for example; the estimation of *θ_K_* was relatively smaller than that of *θ_π_* (Fig. S12). A previous study also reported significant positive Tajima's *D* statistics in populations of European ancestry [48], which might suggest a balancing selection in EUR.

## MATERIALS AND METHODS

### Public and published data

Full sequence data from the 1000 Genomes Project Phase III (KGP) (2504 samples) [49], Estonian Biocentre Human Genome Diversity Panel (EGDP) (402 samples) [50], Human Genome Diversity Project (HGDP)–Centre Etude Polymorphism Humain (CEPH) panel (929 samples) [51], Simons Genome Diversity Project (SGDP) (15 Papuan samples) [52], some indigenous South Asian populations (34 samples) [53], Asian genomes (1243 samples) [54], Chinese COVID-19 patients (1229 samples) [14,28,29], genotypic data of ancient samples from a 1240 K data set (Allen Ancient DNA Resource, version 44.3) (5326 samples) [26], archaic genomes (3 samples) [55–57], and summary data from the ‘COVID-19 host genetics initiative’ (COVID19-hg) [31] were included in our analyses. Ancient samples from >0 and <50 000 years before the present, with known geographic locations, were retained for further analysis. These amounted to 5326 samples in total.

The gene region of *ACE2* is accessed from Ensembl (chrX:15579156–15620271) [58]. We collected 15 genes on the X chromosome that were reported to be related to COVID-19 susceptibility (COVID19Genes) (Table S5). Also, 64 genes related to immunity (ImmuneGenes) were determined based on the Kyoto Encyclopedia of Genes and Genomes (KEGG) database [59].

### Ethical statement

The *ACE2* sequence data of Chinese COVID-19 patients were accessed from previous studies [14,28,29] with the approval of the Ministry of Science and Technology of China (Project IDs: 2020BAT0262, 2020BAT0611 and 2021BAT3145). All procedures performed in this study involving human participants were approved by the Biomedical Research Ethics Committee of Shanghai Institute of Nutrition and Health (no. ER-SINH-262005). 

### Statistical and population genetic analysis

Chromosome-wide genetic diversity was estimated within sliding windows of 10 kb in length, advanced by 5 kb by estimators of nucleotide diversity (*θ_π_*) and numbers of segregating sites (*θ_K_*) using publicly available software [60]. Tajima's *D* statistics [27], Fay and Wu's *H* statistics [37,38], *H12* statistics [39,61,62], Fu and Li's *D* statistics [43], and Fu and Li's *F* statistics [43] were calculated in the same way. The empirical *P*-value was estimated by ranking the statistical values of *ACE2* among all the sliding windows on protein-coding genes across the X chromosome. The confounding factors were controlled as in the previous study [42]. The *DHH* test was designed by combining Tajima's *D*, Fay and Wu's *H*, and *H12* statistics as in a previous study [41], and normalized Fay and Wu's *H* was employed [38].

We performed hierarchical clustering and PCA of *ACE2* sequences based on the distance matrix of genetic differences. We defined the HIMs as those with one allele fixed in at least one of the haplogroups and the other in the remaining haplogroups. We did further haplogroup classification for *ACE2* sequences in all data sets based on the identified HIMs. Haplotype networks were constructed using the median-joining method implemented in Network (version 10) [63], followed by the maximum parsimony method. Within-group TMRCA and cross-group divergence time were estimated on the gene region covered by HIMs (34 kb in length). We performed 100 replicates in total, with 20 sequences randomly sampled from each group for each replicate.

We applied Relate (v1.1.6) [44] to the KGP data set for selection detection. The main procedure of Relate was performed on the combined data set of the X chromosome including all of the 3775 sequences, followed by the ‘EstimatePopulationSize’ and ‘DetectSelection’ analysis for each population. The selection coefficient was estimated based on the allele frequency change inferred by Relate. We assumed that the allele frequency change between every two time points could only be influenced by a constant selection force.

Detailed descriptions of the methods are available in the Supplementary Data.

## DATA AVAILABILITY

Software for calculating genetic diversity as well as other sliding-window-based statistics is available at https://github.com/Shuhua-Group/Theta_D_H.Est/. Software for calculating TMRCA is available at https://github.com/Shuhua-Group/TMRCA/.

## Supplementary Material

nwac118_Supplemental_File

## References

[1] Li W , Moore MJ, Vasilieva N et al. Angiotensin-converting enzyme 2 is a functional receptor for the SARS coronavirus. Nature 2003; 426: 450–4. 10.1038/nature02145 14647384 PMC7095016

[2] Wang Q , Zhang Y, Wu L et al. Structural and functional basis of SARS-CoV-2 entry by using human ACE2. Cell 2020; 181: 894–904. 10.1016/j.cell.2020.03.045 32275855 PMC7144619

[3] Yan R , Zhang Y, Li Y et al. Structural basis for the recognition of SARS-CoV-2 by full-length human ACE2. Science 2020; 367: 1444–8. 10.1126/science.abb2762 32132184 PMC7164635

[4] Letko M , Marzi A, Munster V. Functional assessment of cell entry and receptor usage for SARS-CoV-2 and other lineage B betacoronaviruses. Nat Microbiol 2020; 5: 562–9. 10.1038/s41564-020-0688-y 32094589 PMC7095430

[5] Aguet F , Barbeira AN, Bonazzola R et al. The GTEx Consortium atlas of genetic regulatory effects across human tissues. Science 2020; 369: 562–9. 10.1126/science.aaz1776 PMC7737656 32913098

[6] Ortiz-Fernández L , Sawalha AH. Genetic variability in the expression of the SARS-CoV-2 host cell entry factors across populations. Genes Immun 2020; 21: 269–72. 32759995 10.1038/s41435-020-0107-7 PMC7484169

[7] Santos NPC , Khayat AS, Rodrigues JCG et al. TMPRSS2 variants and their susceptibility to COVID-19: focus in East Asian and European populations. medRxiv 2020.

[8] Chen Y , Shan K, Qian W. Asians do not exhibit elevated expression or unique genetic polymorphisms for ACE2, the cell-entry receptor of SARS-CoV-2. Preprints 2020, 2020020258 (doi: 10.20944/preprints202002.0258.v2).

[9] Cao Y , Li L, Feng Z et al. Comparative genetic analysis of the novel coronavirus (2019-nCoV/SARS-CoV-2) receptor ACE2 in different populations. Cell Discov 2020; 6: 11. 10.1038/s41421-020-0147-1 32133153 PMC7040011

[10] Hou Y , Zhao J, Martin W et al. New insights into genetic susceptibility of COVID-19: an ACE2 and TMPRSS2 polymorphism analysis. BMC Med 2020; 18: 1–8. 10.1186/s12916-020-01673-z 32664879 PMC7360473

[11] Pairo-Castineira E , Clohisey S, Klaric L et al. Genetic mechanisms of critical illness in COVID-19. Nature 2021; 591: 92–8. 33307546 10.1038/s41586-020-03065-y

[12] Ma Y , Huang Y, Zhao S et al. Integrative genomics analysis reveals a 21q22.11 locus contributing risk to COVID-19. Hum Mol Genet 2021; 30: 1247–58. 10.1093/hmg/ddab125 33949668 PMC8136003

[13] Severe Covid-19 GWAS Group . Genomewide association study of severe Covid-19 with respiratory failure. N Engl J Med 2020; 383: 1522–34. 32558485 10.1056/NEJMoa2020283 PMC7315890

[14] Wang F , Huang S, Gao R et al. Initial whole-genome sequencing and analysis of the host genetic contribution to COVID-19 severity and susceptibility. Cell Discov 2020; 6: 83. 10.1038/s41421-020-00231-4 33298875 PMC7653987

[15] Li Y , Ke Y, Xia X et al. Genome-wide association study of COVID-19 severity among the Chinese population. Cell Discov 2021; 7: 76. 10.1038/s41421-021-00318-6 34465742 PMC8408196

[16] Srivastava A , Pandey RK, Singh PP et al. Most frequent South Asian haplotypes of ACE2 share identity by descent with East Eurasian populations. PLoS One 2020; 15: e0238255. 10.1371/journal.pone.0238255 32936832 PMC7494073

[17] Tikellis C , Thomas MC. Angiotensin-converting enzyme 2 (ACE2) is a key modulator of the renin angiotensin system in health and disease. Int J Pept 2012; 2012: 256294. 22536270 10.1155/2012/256294 PMC3321295

[18] Crackower MA , Sarao R, Oudit GY et al. Angiotensin-converting enzyme 2 is an essential regulator of heart function. Nature 2002; 417: 822–8. 10.1038/nature00786 12075344

[19] Benjafield AV , Wang WYS, Morris BJ. No association of angiotensin-converting enzyme 2 gene (ACE2) polymorphisms with essential hypertension. Am J Hypertens 2004; 17: 624–8. 10.1016/j.amjhyper.2004.02.022 15233982 PMC7110370

[20] Lieb W , Graf J, Gotz A et al. Association of angiotensin-converting enzyme 2 (ACE2) gene polymorphisms with parameters of left ventricular hypertrophy in men - Results of the MONICA Augsburg echocardiographic substudy. J Mol Med 2006; 84: 88–96. 10.1007/s00109-005-0718-5 16283142

[21] Zhong J , Yan Z, Liu D et al. Association of angiotensin-converting enzyme 2 gene A/G polymorphism and elevated blood pressure in Chinese patients with metabolic syndrome. J Lab Clin Med 2006; 147: 91–5. 10.1016/j.lab.2005.10.001 16459167 PMC7127450

[22] Huang W , Yang W, Wang Y et al. Association study of angiotensin-converting enzyme 2 gene (ACE2) polymorphisms and essential hypertension in northern Han Chinese. J Hum Hypertens 2006; 20: 968–71. 10.1038/sj.jhh.1002090 17024138

[23] Fan Z , Wu G, Yue M et al. Hypertension and hypertensive left ventricular hypertrophy are associated with ACE2 genetic polymorphism. Life Sci 2019; 225: 39–45. 10.1016/j.lfs.2019.03.059 30917908

[24] Chu JY , Kaliwal Y, Koh M et al. Covid-19 and its cardiac and neurological complications among Ontario visible minorities. Can J Neurol Sci 2021; 49: 504–13. 34162448 10.1017/cjn.2021.148 PMC8365110

[25] Yuan K , Ni X, Liu C et al. Refining models of archaic admixture in Eurasia with ArchaicSeeker 2.0. Nat Commun 2021; 12: 6232. 10.1038/s41467-021-26503-5 34716342 PMC8556419

[26] Mathieson I , Lazaridis I, Rohland N et al. Genome-wide patterns of selection in 230 ancient Eurasians. Nature 2015; 528: 499–503. 10.1038/nature16152 26595274 PMC4918750

[27] Tajima F . Statistical-method for testing the neutral mutation hypothesis by DNA polymorphism. Genetics 1989; 123: 585–95. 10.1093/genetics/123.3.585 2513255 PMC1203831

[28] Wu P , Chen D, Ding W et al. The trans-omics landscape of COVID-19. Nat Commun 2021; 12: 4543. 10.1038/s41467-021-24482-1 34315889 PMC8316550

[29] Zhu H , Zheng F, Li L et al. A Chinese host genetic study discovered IFNs and causality of laboratory traits on COVID-19 severity. iScience 2021; 24: 103186. 34608450 10.1016/j.isci.2021.103186 PMC8481128

[30] Peckham H , de Gruijter NM, Raine C et al. Male sex identified by global COVID-19 meta-analysis as a risk factor for death and ITU admission. Nat Commun 2020; 11: 6317. 10.1038/s41467-020-19741-6 33298944 PMC7726563

[31] Covid 19 Host Genetics Initiative . The COVID-19 Host Genetics Initiative, a global initiative to elucidate the role of host genetic factors in susceptibility and severity of the SARS-CoV-2 virus pandemic. Eur J Hum Genet 2020; 28: 715–18. 10.1038/s41431-020-0636-6 32404885 PMC7220587

[32] Yang Z , Macdonald-Dunlop E, Chen J et al. Genetic landscape of the ACE2 coronavirus receptor. Circulation 2022; 145: 1398–411. 10.1161/CIRCULATIONAHA.121.057888 35387486 PMC9047645

[33] Vangjeli C , Dicker P, Tregouet D-A et al. A polymorphism in ACE2 is associated with a lower risk for fatal cardiovascular events in females: the MORGAM project. J Renin Angiotensin Aldosterone Syst 2011; 12: 504–9. 10.1177/1470320311405557 21490025

[34] Chen Y , Zhang P, Zhou X et al. Relationship between genetic variants of ACE2 gene and circulating levels of ACE2 and its metabolites. J Clin Pharm Ther 2018; 43: 189–95. 10.1111/jcpt.12625 28895159

[35] Zhao Q , Hixson JE, Rao DC et al. Genetic variants in the apelin system and blood pressure responses to dietary sodium interventions: a family-based association study. J Hypertens 2010; 28: 756–63. 10.1097/HJH.0b013e3283370d32 20125035 PMC2905479

[36] Liu D , Chen Y, Zhang P et al. Association between circulating levels of ACE2-Ang-(1-7)-MAS axis and ACE2 gene polymorphisms in hypertensive patients. Medicine (Baltimore) 2016; 95: e3876. 10.1097/MD.0000000000003876 27310975 PMC4998461

[37] Fay JC , Wu CI. Hitchhiking under positive Darwinian selection. Genetics 2000; 155: 1405–13. 10.1093/genetics/155.3.1405 10880498 PMC1461156

[38] Zeng K , Fu YX, Shi SH et al. Statistical tests for detecting positive selection by utilizing high-frequency variants. Genetics 2006; 174: 1431–9. 10.1534/genetics.106.061432 16951063 PMC1667063

[39] Garud NR , Messer PW, Buzbas EO et al. Recent selective sweeps in North American Drosophila melanogaster show signatures of soft sweeps. PLoS Genet 2015; 11: 32. 10.1371/journal.pgen.1005004 PMC4338236 25706129

[40] Zeng K , Mano SH, Shi SH et al. Comparisons of site- and haplotype-frequency methods for detecting positive selection. Mol Biol Evol 2007; 24: 1562–74. 10.1093/molbev/msm078 17449894

[41] Zeng K , Shi S, Wut CI. Compound tests for the detection of hitchhiking under positive selection. Mol Biol Evol 2007; 24: 1898–908. 10.1093/molbev/msm119 17557886

[42] Souilmi Y , Lauterbur ME, Tobler R et al. An ancient viral epidemic involving host coronavirus interacting genes more than 20,000 years ago in East Asia. Curr Biol 2021; 31: 3504–14. 10.1016/j.cub.2021.05.067 34171302 PMC8223470

[43] Fu YX , Li WH. Statistical tests of neutrality of mutations. Genetics 1993; 133: 693–709. 10.1093/genetics/133.3.693 8454210 PMC1205353

[44] Speidel L , Forest M, Shi S et al. A method for genome-wide genealogy estimation for thousands of samples. Nat Genet 2019; 51: 1321–9. 10.1038/s41588-019-0484-x 31477933 PMC7610517

[45] Akbari A , Vitti JJ, Iranmehr A et al. Identifying the favored mutation in a positive selective sweep. Nat Methods 2018; 15: 279–82. 10.1038/nmeth.4606 29457793 PMC6231406

[46] Bhattacharjee MJ , Lin J-J, Chang C-Y et al. Identifying primate ACE2 variants that confer resistance to SARS-CoV-2. Mol Biol Evol 2021; 38: 2715–31. 10.1093/molbev/msab060 33674876 PMC7989403

[47] Fam BS , Vargas-Pinilla P, Amorim CEG et al. ACE2 diversity in placental mammals reveals the evolutionary strategy of SARS-CoV-2. Genet Mol Biol 2020; 43: e20200104. 10.1590/1678-4685-gmb-2020-0104 32520981 PMC7278419

[48] Akey JM , Eberle MA, Rieder MJ et al. Population history and natural selection shape patterns of genetic variation in 132 genes. PLoS Biol 2004; 2: 2715–31. 10.1371/journal.pbio.0020286 PMC515367 15361935

[49] Altshuler DM , Durbin RM, Abecasis GR et al. A global reference for human genetic variation. Nature 2015; 526: 68–74. 26432245 10.1038/nature15393 PMC4750478

[50] Pagani L , Lawson DJ, Jagoda E et al. Genomic analyses inform on migration events during the peopling of Eurasia. Nature 2016; 538: 238–42. 10.1038/nature19792 27654910 PMC5164938

[51] Bergström A , McCarthy SA, Hui R et al. Insights into human genetic variation and population history from 929 diverse genomes. Science 2020; 367: eaay5012. 10.1126/science.aay5012 32193295 PMC7115999

[52] Mallick S , Li H, Lipson M et al. The Simons genome diversity project: 300 genomes from 142 diverse populations. Nature 2016; 538: 201–6. 10.1038/nature18964 27654912 PMC5161557

[53] Mondal M , Casals F, Xu T et al. Genomic analysis of Andamanese provides insights into ancient human migration into Asia and adaptation. Nat Genet 2016; 48: 1066–70. 10.1038/ng.3621 27455350

[54] Zhang C , Gao Y, Ning Z et al. PGG.SNV: understanding the evolutionary and medical implications of human single nucleotide variations in diverse populations. Genome Biol 2019; 20: 215. 10.1186/s13059-019-1838-5 31640808 PMC6805450

[55] Prufer K , Racimo F, Patterson N et al. The complete genome sequence of a Neanderthal from the Altai Mountains. Nature 2014; 505: 43–9. 10.1038/nature12886 24352235 PMC4031459

[56] Prufer K , de Filippo C, Grote S et al. A high-coverage Neanderthal genome from Vindija Cave in Croatia. Science 2017; 358: 655–8. 10.1126/science.aao1887 28982794 PMC6185897

[57] Meyer M , Kircher M, Gansauge MT et al. A high-coverage genome sequence from an Archaic Denisovan Individual. Science 2012; 338: 222–6. 10.1126/science.1224344 22936568 PMC3617501

[58] Howe KL , Achuthan P, Allen J et al. Ensembl 2021. Nucleic Acids Res 2021; 49: D884–91. 10.1093/nar/gkaa942 33137190 PMC7778975

[59] Kanehisa M , Furumichi M, Tanabe M et al. KEGG: new perspectives on genomes, pathways, diseases and drugs. Nucleic Acids Res 2017; 45: D353–61. 10.1093/nar/gkw1092 27899662 PMC5210567

[60] Pan YW , Zhang C, Lu Y et al. Genomic diversity and post-admixture adaptation in the Uyghurs. Natl Sci Rev 2022; 9: nwab124. 10.1093/nsr/nwab124 35350227 PMC8953455

[61] Harris AM , DeGiorgio M. Identifying and classifying shared selective sweeps from multilocus data. Genetics 2020; 215: 143–71. 10.1534/genetics.120.303137 32152048 PMC7198270

[62] Harris AM , Garud NR, DeGiorgio M. Detection and classification of hard and soft sweeps from unphased genotypes by multilocus genotype identity. Genetics 2018; 210: 1429–52. 10.1534/genetics.118.301502 30315068 PMC6283157

[63] Bandelt HJ , Forster P, Rohl A. Median-joining networks for inferring intraspecific phylogenies. Mol Biol Evol 1999; 16: 37–48. 10.1093/oxfordjournals.molbev.a026036 10331250

