# Supplementary material for: Lineage-specific positive selection on ACE2 contributes to the genetic susceptibility of COVID-19
Source: Natl Sci Rev. 2022 Jul 1;9(9):nwac118. doi: 10.1093/nsr/nwac118 (PMC9529360; doi:10.1093/nsr/nwac118)
Supplement: nwac118_Supplemental_File [file nwac118_supplemental_file.pdf]

# Supplementary Materials

## Lineage-specific positive selection on *ACE2* contributes to the genetic susceptibility of COVID-19

Yuwen Pan, Panhong Liu, Fang Wang, Peng Wu, Fanjun Cheng, Xin Jin, Shuhua Xu*

*Correspondence and requests for materials should be addressed to S.X. (Email: xushua@fudan.edu.cn).

## Text S1. Data quality of the *ACE2* region

We evaluated the data quality of the *ACE2* sequences in our analyses based on the genome masks (http://ftp.1000genomes.ebi.ac.uk/vol1/ftp/release/20130502/supporting/accessible_genome_masks/) from the 1000 Genomes Project. There were 2 different types of masks, pilot and strict, and both were used. For the pilot mask, sites with a depth of coverage of <8960 or >35840 across all samples were excluded; for the strict mask, sites with a depth of coverage of <8960 or >26880 were excluded. Overall, 91.9% bases on the X chromosome passed all filters according to the pilot mask, and 70.7% passed under the strict mask. For the *ACE2* region, the proportions are 99.5% and 91.7% based on the pilot and strict mask, respectively. These summary statistics indicate the high quality of the *ACE2* region compared with the rest of the X chromosome. For the 39 HIMs, only 5 markers failed the strict mask, with one carrying the haplogroup-specific allele for *ACE2*-hg3 and the other 4 related to *ACE2*-hg4. Since there are 8 *ACE2*-hg3-specific makers and 12 *ACE2*-hg4-specific makers among the 39 HIMs, the relatively lower quality of the 5 markers wouldn't influence the *ACE2*-hg inference.

We further estimated the $r^2$ measure of the pairwise linkage among HIMs for each population using vcftools (1). Strong linkage disequilibrium was observed among the HIMs (Fig. S1.1). Genetic maps of HapMap (phase II) and African American (2) were also inspected. The HapMap genetic map was generated by LDhat (3) and should reflect the average recombination rates across time. There are 2 loci with moderate recombination rate, respectively reaching 8 cM/Mb and 4 cM/Mb (Fig. S1.2A), which would indicate the ancient recombination events. The genetic map of African American was inferred based on the local ancestry and could reflect the recombination events in the recent generations. Low recombination rates were observed within the *ACE2* region (<1 cM/Mb) (Fig. S1.2B). The genetic map of deCODE (4) was also accessed but not analyzed due to the low coverage of variants in the *ACE2* region.

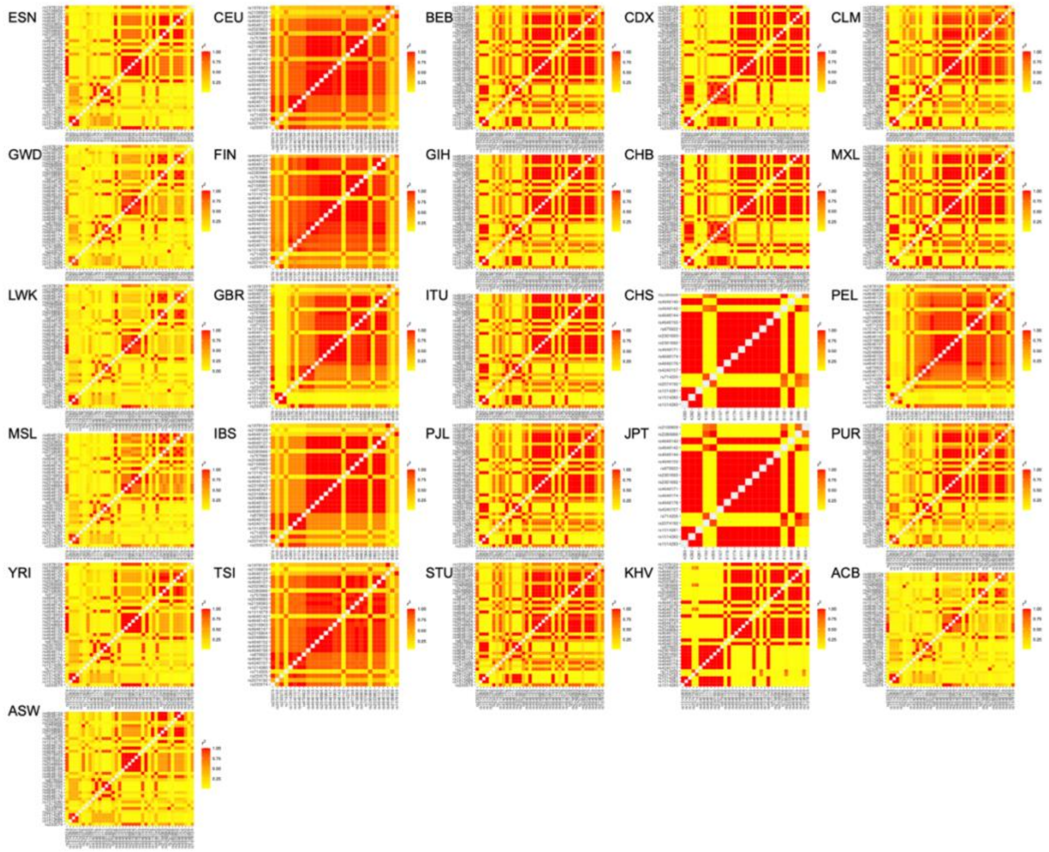


**Figure S1.1 | Heatmap of the pairwise $r^2$ among HIMs.**

Only segregating sites were presented in the plot.

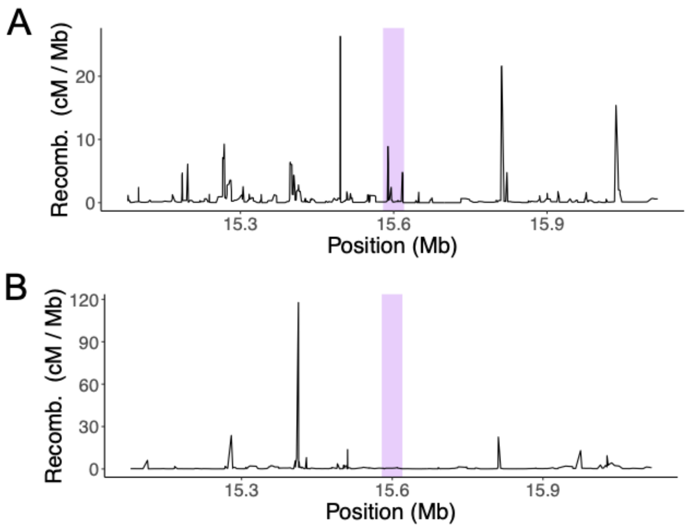


**Figure S1.2 | Recombination rate of the *ACE2* region (in purple).**

**A.** Genetic map of HapMap; **B.** Genetic map of African American.

## Text S2. Association analysis based on female samples

We found that females tended to be mild, while males were likely to have higher severity (Odds Ratio (OR) = 1.40, $P < 1.44\times10^{-3}$). When individuals were grouped more broadly, i.e., asymptomatic, mild, moderate severities, and others, the pattern was retained (OR = 1.43, $P < 2.07\times10^{-3}$). It was in agreement with the previous findings (5). There should be some protective mechanism for females. When it turns for the association analysis based on female samples, no significant signal was detected between COVID-19 severities and haplogroup types (Fig. S2.1).

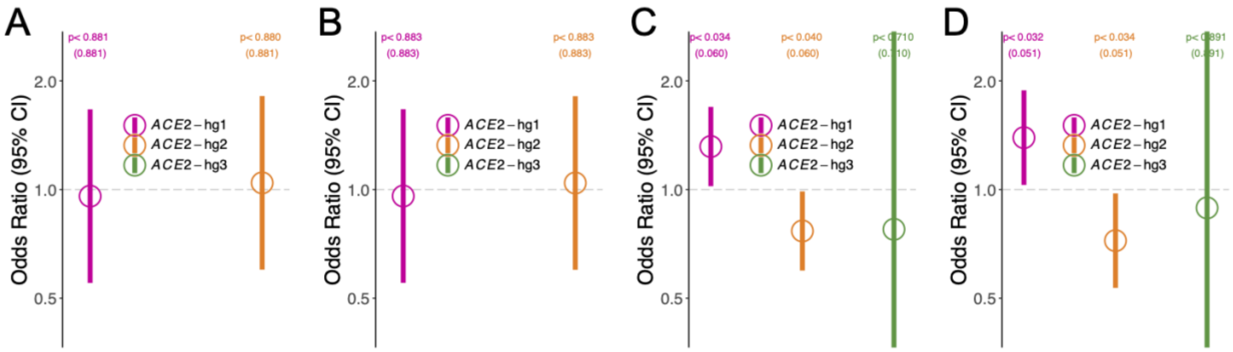


**Figure S2.1 | Association studies of COVID-19 severities with *ACE2* haplogroups based on female samples.**

**A.** Ordered logistic regression between the COVID-19 severities and haplogroup type for female samples; **B.** Logistic regression between the COVID-19 severities (broad group) and haplogroup type for female samples; **C.** Ordered logistic regression between the COVID-19 severities and haplogroup using both male and female samples; **D.** Logistic regression between the COVID-19 severities (broad group) and haplogroup type using both male and female samples. Only haplotype-homozygous females (n=308) were employed in the analyses to eliminate the potential bias caused by the interaction of different haplogroups in the same female individuals. Age, sex, comorbidities (whether diagnosed or not), and ancestry (the top 5 principal components) were used as covariates in the association analyses. *P* values in the bracket were BH-corrected.

## Text S3. Detailed methods

### Public and Published Data

Full sequence data from the 1000 Genomes Project Phase III (KGP) (2,504 samples) (6), Estonian Biocentre Human Genome Diversity Panel (EGDP) (402 samples) (7), Human Genome Diversity Project (HGDP)-CEPH panel (929 samples) (8), Simons Genome Diversity Project (SGDP) (15 Papuan samples) (9), some indigenous South Asian populations (34 samples) (10), Asian genomes (1,243 samples) (11), Chinese COVID-19 patients (1,229 samples) (12-14), genotypic data of ancient samples from 1240K dataset (Allen Ancient DNA Resource, https://reich.hms.harvard.edu/allen-ancient-dna-resource-aadr-downloadable-genotypes-present-day-and-ancient-dna-data, version 44.3) (5,326 samples) (15), archaic genomes (3 samples) (16-18), and summary data from "COVID-19 host genetics initiative" (COVID19-hg) (https://www.covid19hg.org/) (19) were included in our analyses. Ancient samples of date >0 and <50,000 years before present and with the known geographic location were retained for further analysis, including 5,326 samples in total. The ancestral sequence was downloaded from the 1000 Genomes database (e71) to determine the ancestral and derived alleles for SNVs in our analyses. Coordinates of variants in the HGDP dataset were lifted over to build 37 using Picard toolkit version 1.117 (http://broadinstitute.github.io/picard/). Phasing of the X chromosomes in 1240K and HGDP datasets was performed using SHAPEIT2 (20) for haplogroup inference, and variants and samples with a missing rate >20% were removed before phasing. The gene region of *ACE2* is accessed from Ensembl (chrX:15,579,156-15,620,271) (21).

### Estimation of genetic diversity, Tajima's *D*, Fay & Wu's *H*, *H12*, Fu & Li's *D,* Fu & Li's *F,* and *DHH* statistics

The chromosome-wide genetic diversity was estimated within sliding windows of 10 kb in length advanced by 5 kb by estimators of nucleotide diversity ($\theta_\pi$) and numbers of segregating sites ($\theta_K$). The Tajima's *D* statistics (22), Fay & Wu's *H* statistics (23, 24), *H12* statistics (25-27), Fu & Li's *D* statistics (28), and Fu & Li's *F* statistics (28) were calculated in the same way. The theoretical *P*-value of Tajima's *D* statistics was estimated following the $\beta$ distribution as instructed in the reference paper. The empirical *P*-value was estimated by ranking the statistical values of *ACE2* among all the sliding windows on protein-coding genes across the X chromosome. The confounding factors were controlled following the previous study (29), including the density of

conserved segments, the density of regulatory elements, density of coding sequence, GC content, and recombination rate. Any windows containing less than 10 segregating sites were dropped in our analysis. 100 sequences were randomly sampled from the KGP dataset for each population to balance the sample size.

The *DHH* test was designed by combining Tajima's *D*, Fay & Wu's *H*, and *H12* statistics following a previous study (30). Normalized Fay & Wu's *H* was employed (24). We denote the observed values of Tajima's *D*, Fay & Wu's *H*, and *H12* statistics in a given window as *D'*, *H'*, and *H12'*. These observed values were rejected by the *DHH* test at the significance level *P* if

$$D' \leq D_{cri},\ H' \leq H_{cri},\ \text{and}\ H12' \leq H12_{cri}$$

where $D_{cri}$, $H_{cri}$, and $H12_{cri}$ are critical values satisfying

$$P(D' \leq D_{cri},\ H' \leq H_{cri},\ \text{and}\ H12' \leq H12_{cri}) = P$$

and

$$P(D' \leq D_{cri}) = P(H' \leq H_{cri}) = P(H12' \leq H12_{cri}) = P^{*}$$

## Haplotype clustering and haplogroup determination

The distance matrix of identical-by-state (IBS) was calculated for all *ACE2* sequences (n=3,775) from the KGP dataset. We did hierarchical clustering using the R package "hclust". Tree visualization was achieved by the R package "ggtree" (31). We also constructed the asymmetric distance matrix (*M*) for the *ACE2* sequences, and $M_{ij}$ on *i*th row and the *j*th column was defined as the number of alleles that exist in sequence *i* but not in *j*. We performed principal component analysis (PCA) based on the asymmetric matrix using the R package "prcomp". Then we extracted the sequences in each clustering center and calculated allele frequencies for each group. We defined the haplogroup informative markers (HIMs) as those with one allele fixed in at least one of the haplogroups and the other in the remaining haplogroups. We identified 39 SNVs. We did further haplogroup classification for *ACE2* sequences in all datasets based on the HIMs. The mismatch rate was given as 10%, accepting 3 differences out of the 39 alleles. All the *ACE2* sequences were well classified in our analyses.

## Construction of haplotype networks

Haplotype networks were constructed using Network (version 10) (32). For the global populations, SNVs with AF <0.05 or >0.95 in all of the populations from the KGP dataset were removed from

the haplotype data, remaining 96 SNVs. For the continental groups, all SNVs were used while constructing the haplotype network. For the ancient DNA, variants and samples with a missing rate >20% were removed before phasing, and the remaining 281 *ACE2* sequences and 15 SNVs for the network analysis. The networks were calculated using the median-joining (MJ) method, followed by the maximum parsimony (MP) method.

## Estimation of the time to a most recent common ancestor (TMRCA) and divergence time

We firstly grouped sequences from the same populations and of the same haplogroups, named after their population names and haplogroups (pop-hap). Within-pop-hap TMRCA and cross-pop-hap divergence time were then estimated on the gene region covered by HIMs, which is 34 kb in length and 83% of full *ACE2* length. We performed 100 replicates in total, with 20 sequences randomly sampled from each pop-hap group for each replicate. We calculated the TMRCA for each pop-hap group according to the following formula:

$$TMRCA = \frac{\pi_{TMRCA}}{2 \times \mu \times L}$$

where $\pi_{TMRCA}$ is the pairwise difference and was calculated using the formula below:

$$\pi_{TMRCA} = \sum_{ij} x_i x_j \pi_{ij}$$

in which $x_i$ is the frequency of the *i*th sequence in the proportion and $\pi_{ij}$ is the number of nucleotide differences per nucleotide site between the *i*th and *j*th sequences (33). And $\mu$ is the local mutation rate of the target region with length $L$. It was estimated as

$$\mu = \frac{d_{Human-Chimp}}{2 \times L \times T_{Human-Chimp}}$$

where $d_{Human-Chimp}$ is nucleotide differences between the human reference genome and that of the chimpanzee of the target region. $T_{Human-Chimp}$ denotes the divergence time of humans and chimpanzees, and it was given as 13 million years (34). The genome of the chimpanzee (panTro5) was accessed from UCSC Genome Browser (https://genome.ucsc.edu/).

As for the divergence time, it was estimated using the following formula:

$$Divergence = \frac{\pi_{Divergence}}{2 \times \mu \times L}$$

where $\mu$ is the same as mentioned above and $\pi_{Divergence}$ is the pairwise difference across pop-hap groups. $\pi_{Divergence}$ was calculated as below:

$$\pi_{Divergence} = \sum_{nm} x_n x_m \pi_{nm}$$

in which $x_n$ is the frequency of the $n$th sequence in the proportion and $\pi_{nm}$ is the number of nucleotide differences per nucleotide site between $n$th and $m$th sequences, while the $n$th and $m$th sequences were from different pop-hap groups.

## Selection analysis with Relate

We applied Relate (v1.1.6) (35) to the KGP dataset for selection detection. We treated all sequences as haploids by converting the input file format accordingly. The main procedure of Relate was performed on the combined dataset of the X chromosome including all of the 3775 sequences. We then estimated population sizes for each population separately, with the option "--threshold" set as 0, followed by the "DetectSelection" program. The distribution of population size estimated based on the X chromosome (Fig. S17) was similar to those of autosomes presented in the Relate paper. We used $1.25\times10^{-8}$ per site per generation as the mutation rate (35) and 25 for the number of years per generation (34, 36-38). Similar results were also obtained while using other values of mutation rate (Fig. S18).

## Estimation of selection coefficient

The selection coefficient was estimated based on the allele frequency change inferred by Relate. For a given biallelic SNV (with allele 1/0), we assumed the fitness ($W$) of genotype 1 in males and 1/1 in females equal 1, genotype 0 in males and 1/0 in females equal $1 - s$, and 0/0 in female equals $1 - 2 \times s$ assuming an additive model, where $s$ denotes the selection coefficient. For the dominant model, we assumed the fitness of 1 for genotype 1 (males) as well as genotypes 1/1 and 1/0 (females), and that of $1 - s$ for genotype 0 in males and 0/0 in females. The initial frequencies ($f$) for each genotype were estimated based on the Hardy-Weinberg Equilibrium for the initial allele frequencies ($f_{1\_init}$) inferred by Relate, that is

$$f_{1\_init\_male} = f_{1\_init}$$

$$f_{0\_init\_male} = 1 - f_{1\_init}$$

$$f_{1/1\_init\_female} = f_{1\_init}^2$$

$$f_{0/0\_init\_female} = (1 - f_{1\_init})^2$$

$$f_{1/0\_init\_female} = 2 \times f_{1\_init} \times (1 - f_{1\_init})$$

For a given generation with known genotype frequencies, the genotype frequencies in the next generation ($f'$) could be estimated using the following formulas:

$$\bar{W}_{male} = W_1 \times f_1 + W_0 \times f_0$$

$$\bar{W}_{female} = W_{1/1} \times f_{1/1} + W_{1/0} \times f_{1/0} + W_{0/0} \times f_{0/0}$$

$$f'_{1\_male} = \frac{W_{1/1} \times f_{1/1} + W_{1/0} \times f_{1/0} \times 0.5}{\bar{W}_{female}}$$

$$f'_{0\_male} = \frac{W_{0/0} \times f_{0/0} + W_{1/0} \times f_{1/0} \times 0.5}{\bar{W}_{female}}$$

$$f'_{1/1\_female} = \frac{(W_{1/1} \times f_{1/1} + W_{1/0} \times f_{1/0} \times 0.5) \times (W_1 \times f_1)}{\bar{W}_{female} \times \bar{W}_{male}}$$

$$f'_{0/0\_female} = \frac{(W_{0/0} \times f_{0/0} + W_{1/0} \times f_{1/0} \times 0.5) \times (W_0 \times f_0)}{\bar{W}_{female} \times \bar{W}_{male}}$$

$$f'_{1/0\_female} = 1 - f'_{1/1\_female} - f'_{0/0\_female}$$

so that the allele frequencies in the next generation should be

$$f'_1 = \frac{f'_{1\_male} + 2 \times f'_{1/1\_female} + f'_{1/0\_female}}{3}$$

$$f'_0 = \frac{f'_{0\_male} + 2 \times f'_{0/0\_female} + f'_{1/0\_female}}{3}$$

We assumed the constant value of the selection coefficient between every 2 time points (given by Relate). We tried to get the estimates of the selection coefficient by iteration. We stopped the iterations until the difference between our estimated $f'_1$ and that inferred by Relate less than 0.0001.

## Collection of COVID19Genes

We collected 15 genes on the X chromosome that were reported to be related to COVID-19 susceptibility (COVID19Genes) (Table S5). And 64 genes related to immunity (ImmuneGenes) were determined based on the KEGG database (39), involving pathways “5.1 Immune_system”, “6.3 Immune disease”, “6.8 Infectious disease: bacterial”, and “6.9 Infectious disease: viral”.

# Supplementary Figures

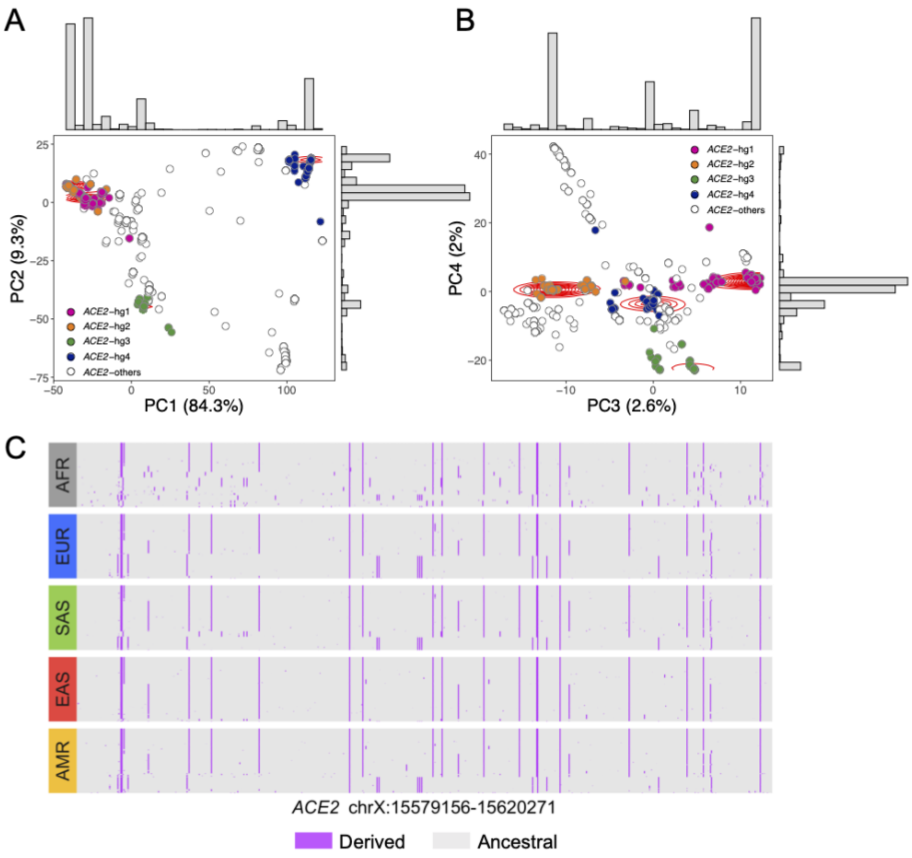


**Figure S1 | Haplotype clustering of the *ACE2* sequences.**

**A.** Principal component analysis of *ACE2* sequences (PC1–PC2). Red circles indicate the clustering centers for a high density of sequences. The histogram on the sidebars indicates the numbers of sequences; **B.** Principal component analysis of *ACE2* sequences (PC3–PC4); **C.** Haplotype plot for *ACE2* sequences. Each column indicates a SNV and each row is a sequence. Ancestral and derived alleles were in different colors.

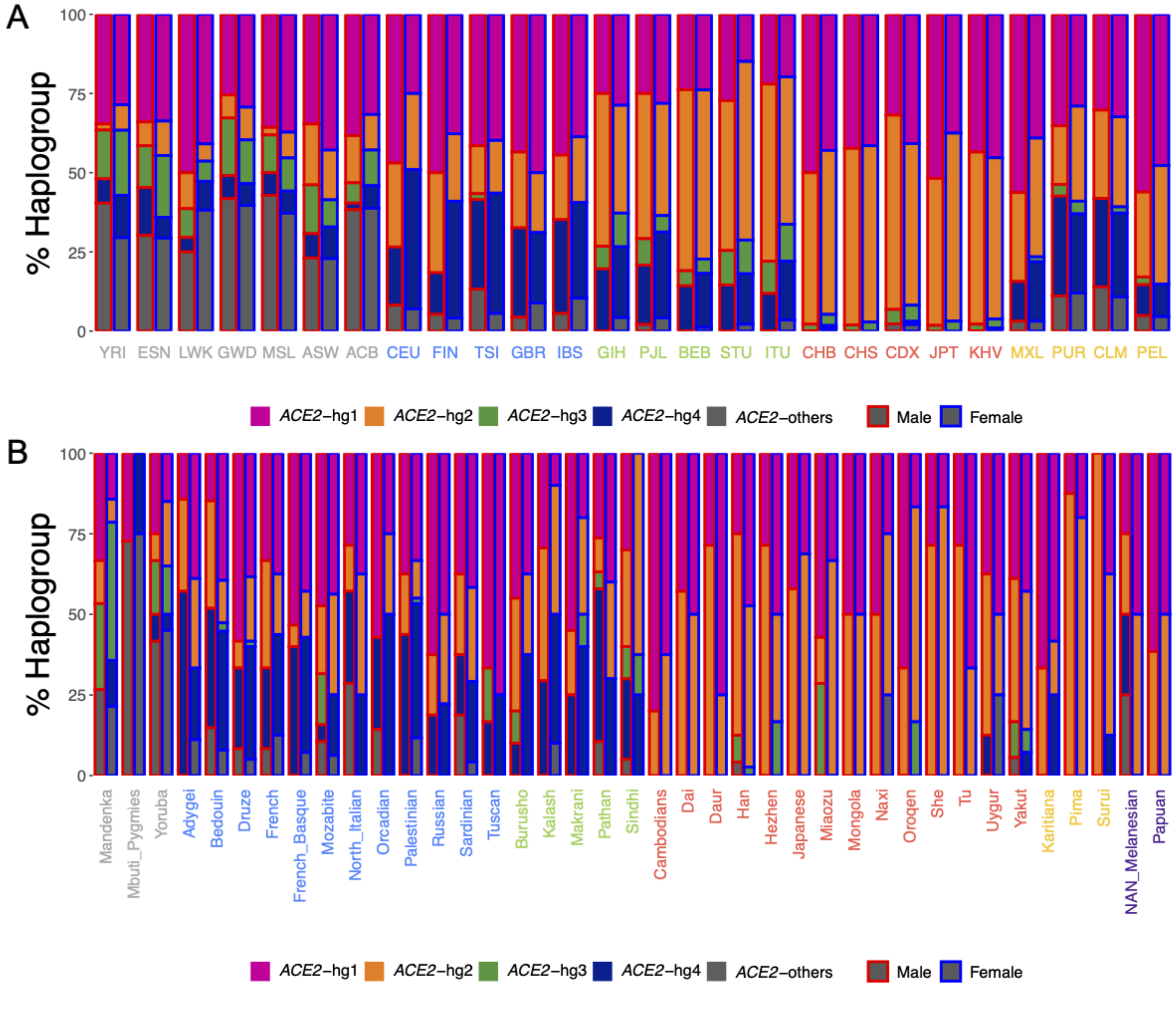


**Figure S2 | Haplogroup frequencies across worldwide populations.**

**A.** Haplogroup frequencies estimated based on the 1000 Genomes Project; **B.** Haplogroup frequencies estimated based on the HGDP dataset.

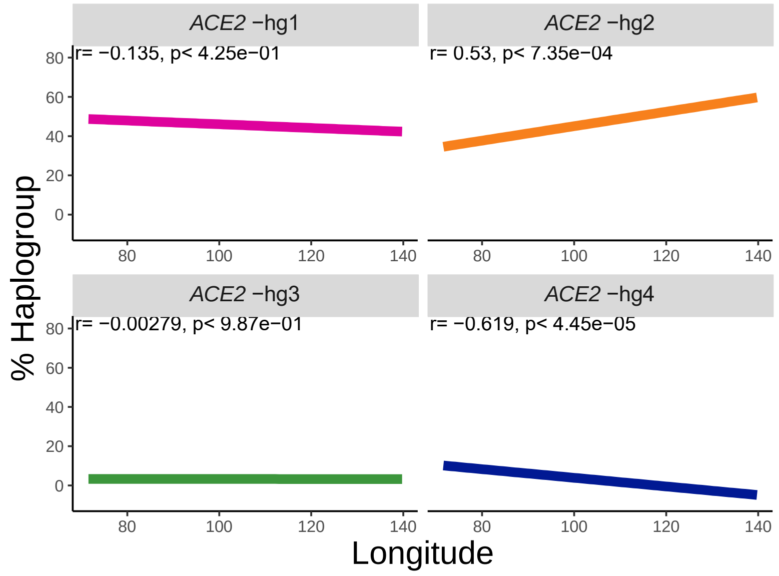


**Figure S3 | Association between haplogroup frequency and longitude in EAS populations.**

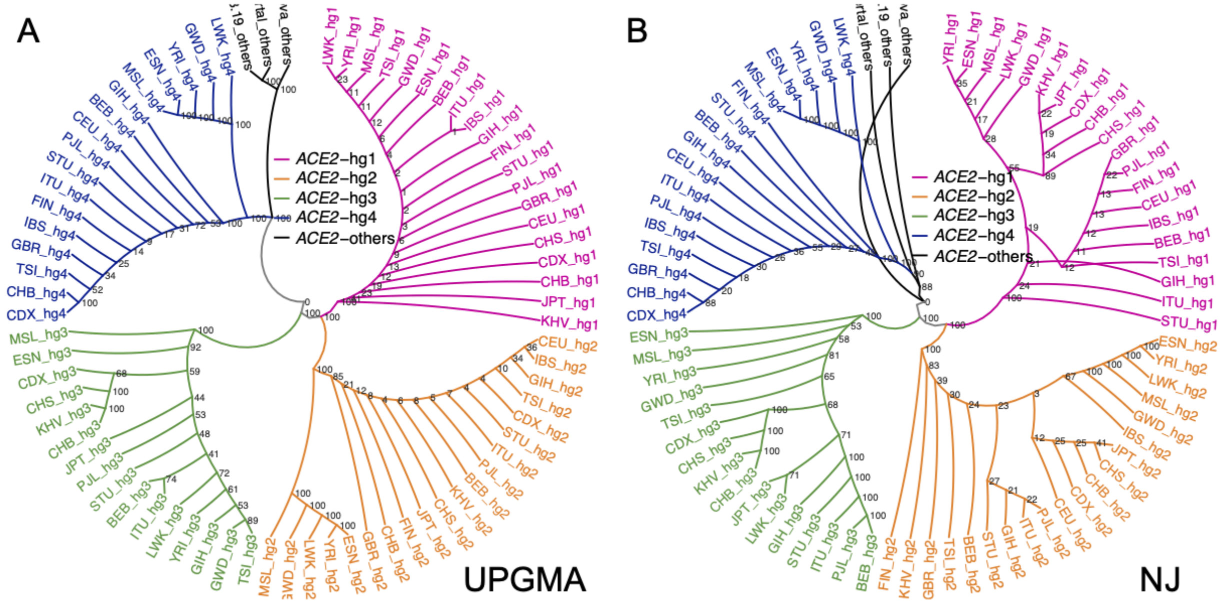


**Figure S4 | Divergence tree of *ACE2* sequences across the worldwide populations.**

**A.** Divergence tree constructed based on the UPGMA algorithm; **B.** Divergence tree constructed based on the NJ algorithm. Sequences from the same populations and of the same haplogroups were grouped together, named after their population names and haplogroups, i.e., pop-hap. We excluded minor haplogroups in modern human populations from this analysis. Sequences of archaic hominids were used as outgroups. We performed 100 times bootstrap by random sampling 20 sequences from each pop-hap group for each replicate. The numbers labeled on the trees were bootstrap values.

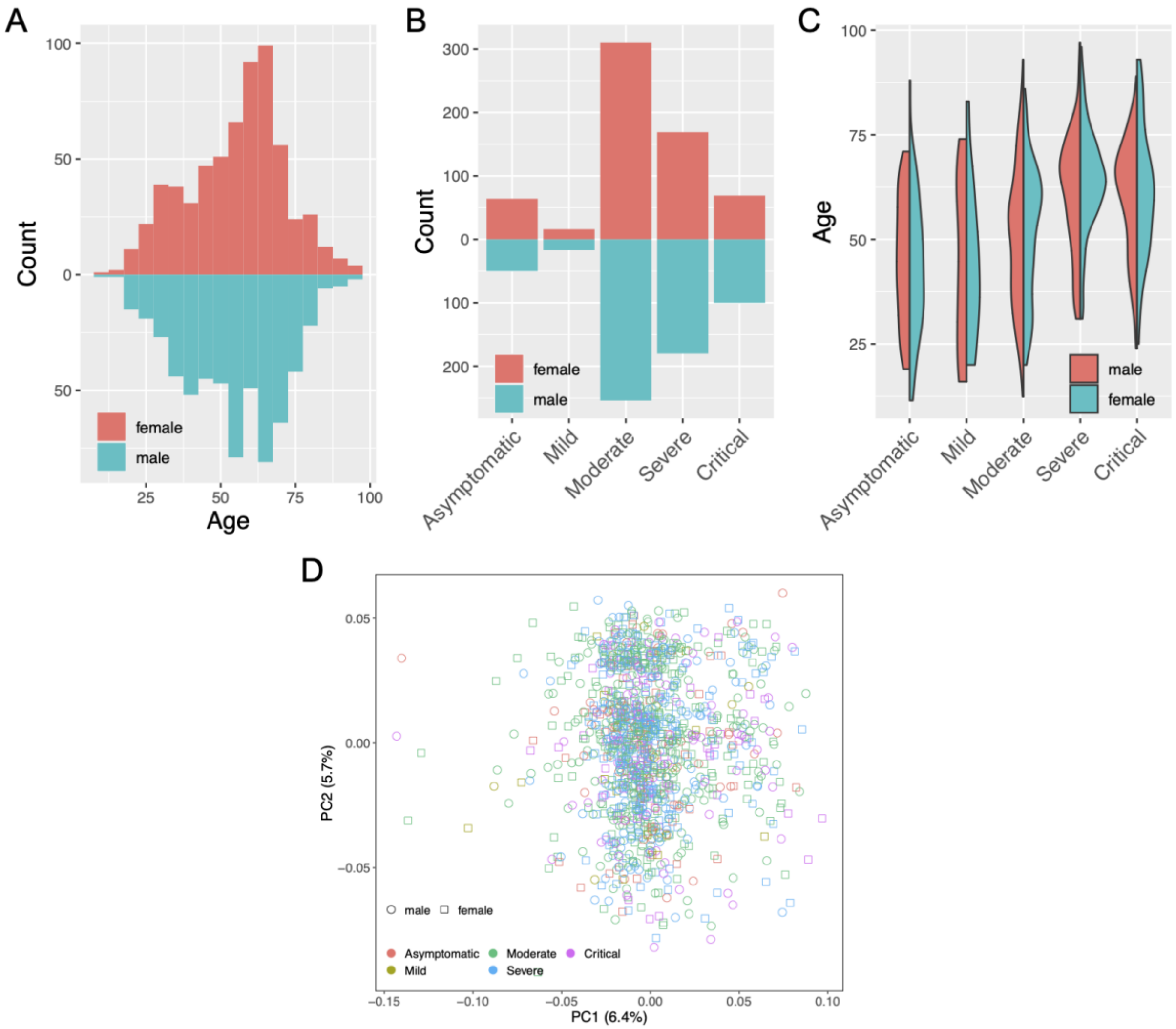


**Figure S5 | Assessments of the recruited COVID-19 patients.**

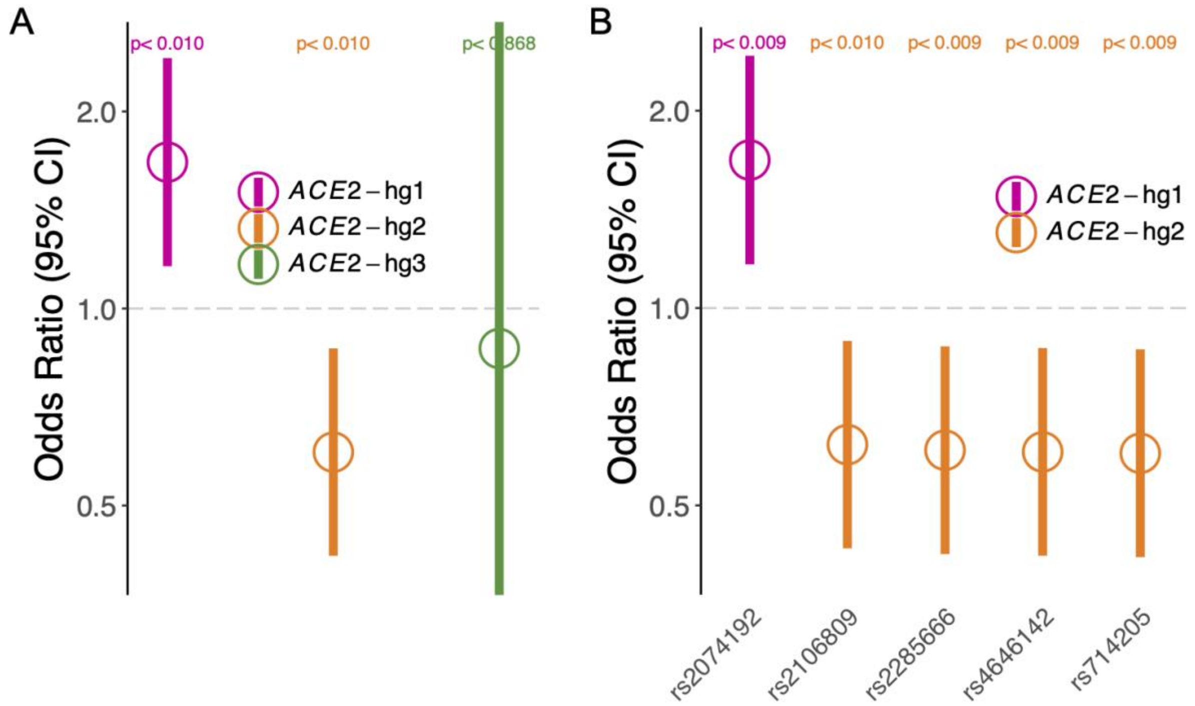


**Figure S6 | Logistic regression between COVID-19 severity and *ACE2* variation.**

**A.** Association between COVID-19 severity and *ACE2* haplogroup type (OR=1.67 for *ACE2*-hg1, OR=0.60 for *ACE2*-hg2); **B.** Association between COVID-19 severity and allele frequency of haplogroup-specific HIMs on *ACE2*-hg1 and *ACE2*-hg2. *P* values labeled on the plot are BH-corrected. Age, comorbidities (whether diagnosed or not), and ancestry (the top 5 principal components) were used as the covariates in the association analyses. OR=1.68, 0.60, 0.60, 0.61, and 0.62 respectively for rs2074192, rs714205, rs4646142, rs2285666, rs2106809.

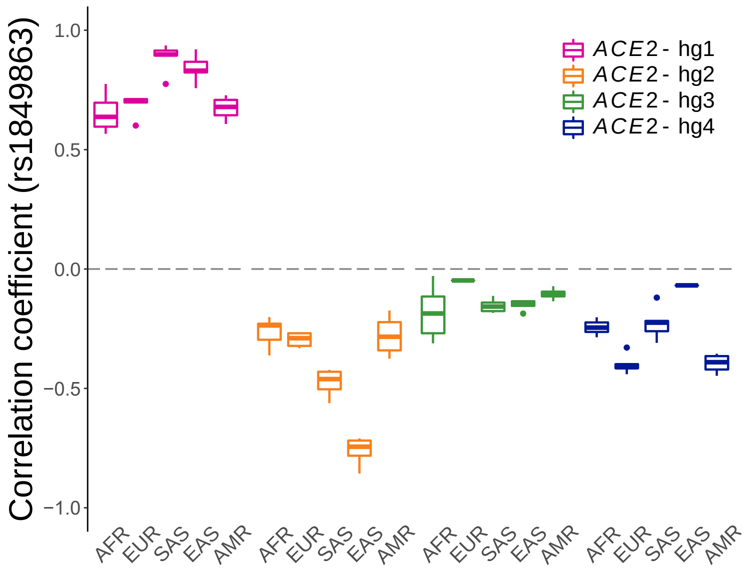


**Figure S7 | Association between *ACE2* haplogroup type and rs1849863-C variation.**

Association analysis was conducted for each haplogroup type in a single population. And all the *ACE2* sequences were used. We estimated the correlation between *ACE2* haplogroup type (is or not a given haplogroup) and rs1849863-C variation (does or not carry the derived allele). Significantly negative correlation was observed between *ACE2*-hg2 and rs1849863-C in EAS, which was due to the dominance of *ACE2*-hg1 and *ACE2*-hg2 in EAS.

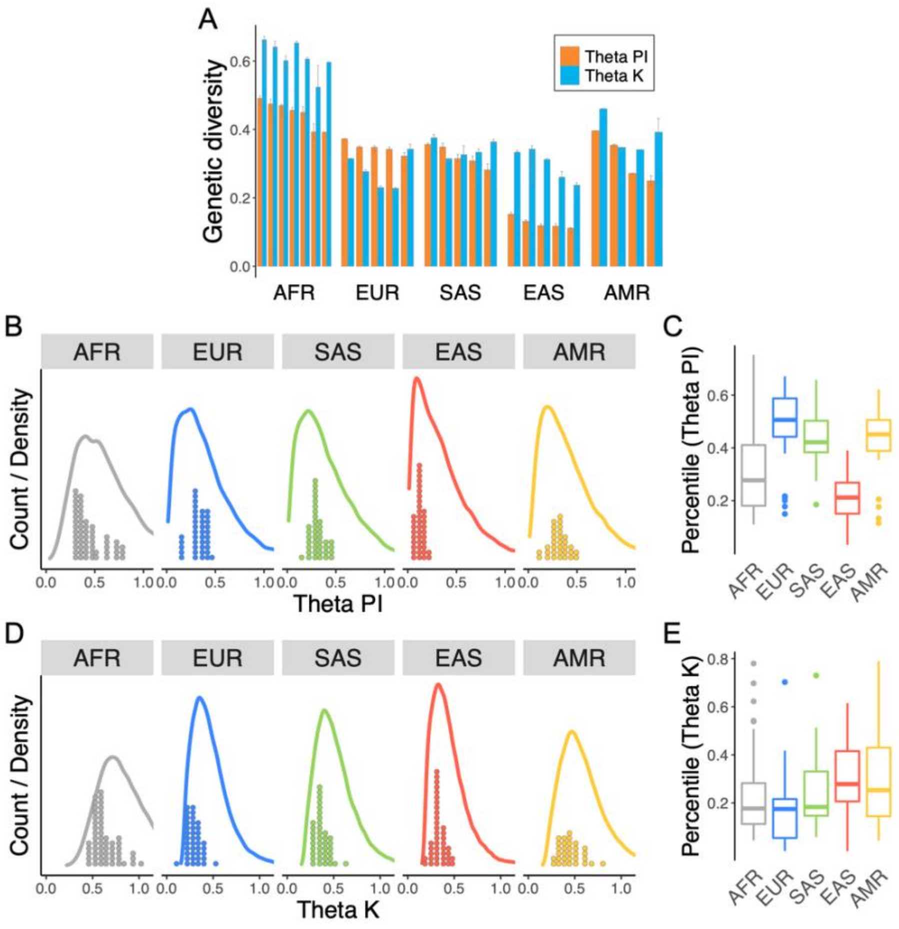


**Figure S8 | Genetic diversity of *ACE2* across the worldwide populations.**

**A.** Genetic diversity estimated for the full-length *ACE2* by the estimators of both nucleotide diversity ($\theta_\pi$) and a number of segregating sites ($\theta_K$). 10 replicates were conducted with 100 sequences randomly sampled for each; **B.** Distributions of the nucleotide diversity of *ACE2* under the X-chromosome-wide background. Each dot denotes the nucleotide diversity of *ACE2* estimated within sliding windows of 10 kb in length by an increment of 5 kb for each population. And the lines are the X-chromosome-wide distribution of diversity estimated in the same way with confounding factors under control. We grouped continental populations due to their similar diversity profiles. And 100 sequences were random sampled from the 1000 Genome Project for each population to balance the sample size; **C.** Percentiles of the *ACE2* nucleotide diversity estimated within sliding windows under the chromosome-wide context. The large percentile indicates the top rank. The result indicates the even lower nucleotide diversity of *ACE2* compared with the X-chromosome-wide background in EAS; **D.** Distributions of the numbers of segregating sites of *ACE2* under the X-chromosome-wide background. The statistical values were estimated and presented in the same way as the nucleotide diversity; **E.** Percentiles of the numbers of segregating sites of *ACE2* estimated within sliding windows under the chromosome-wide context.

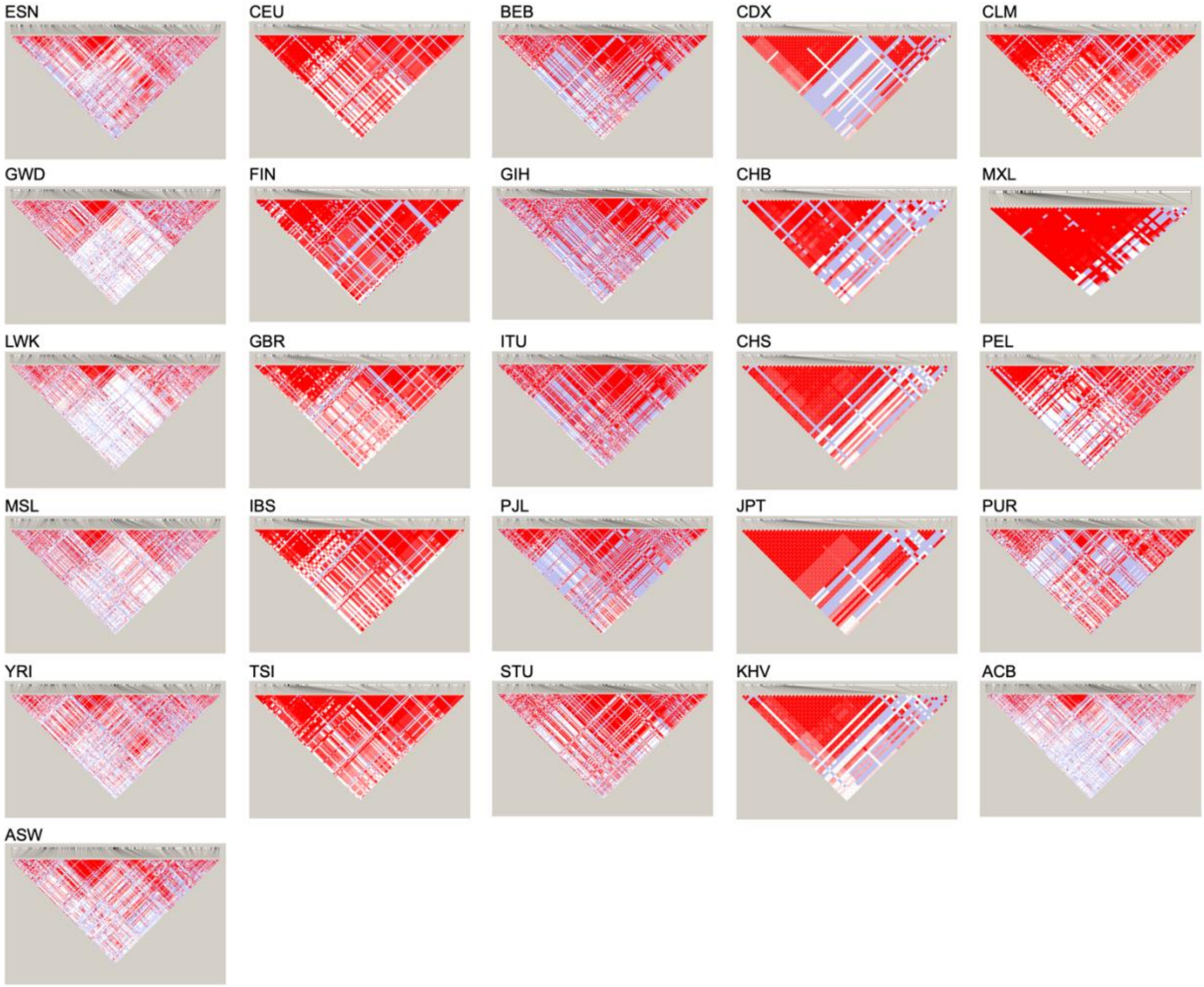


**Figure S9 | Linkage of variants at *ACE2* and *BMX* among the worldwide populations.**

The linkage was estimated as $r^2$ using haploview (40). Randomly sampled 100 sequences for each population to balance the sample size.

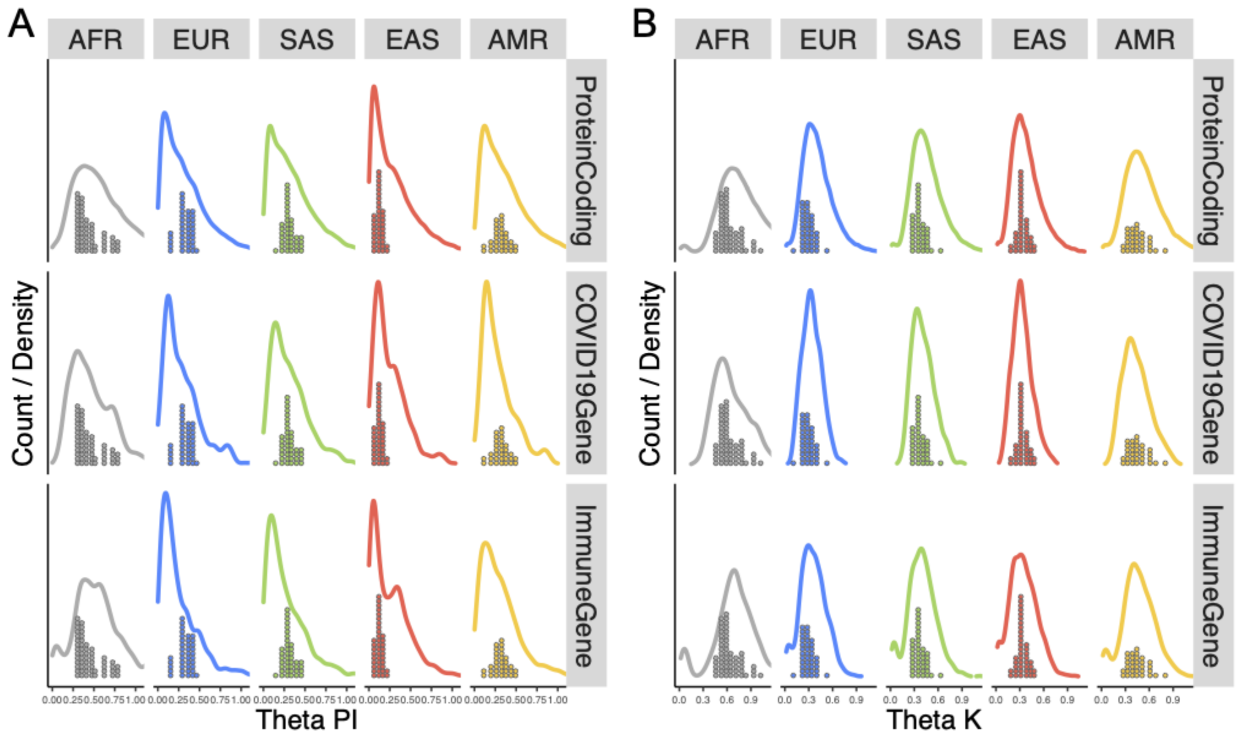


**Figure S10 | Comparisons of genetic diversity between *ACE2* and the reference gene sets.**

Each dot denotes the genetic diversity of *ACE2* estimated within sliding windows of 10 kb in length by an increment of 5 kb for each population. And the lines are the distributions of genetic diversity estimated for the protein-coding genes, COVID19Genes, and ImmuneGenes on the X chromosome. We grouped continental populations due to their similar profiles of genetic diversity.

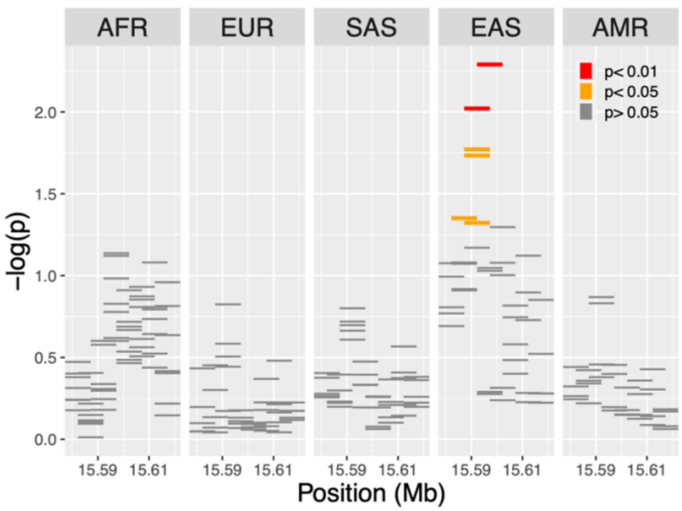


**Figure S11 | Statistical significance of the *DHH* test on *ACE2*.**

Each line represents the *P*-value of the *DHH* statistics estimated within a specific window in one single population.

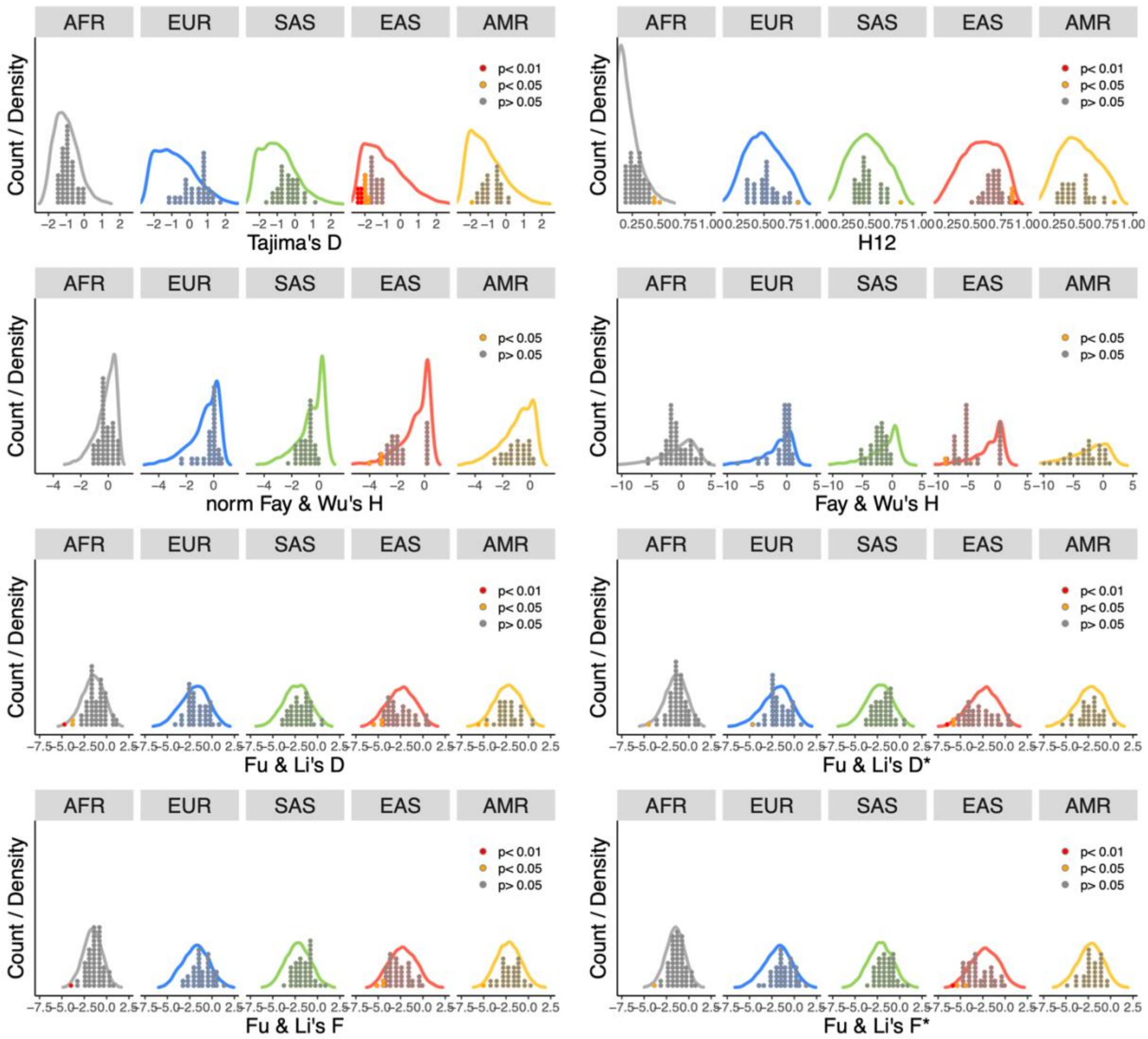


**Figure S12 | Signals of natural selection on *ACE2*.**

Distributions of the Tajima's *D*, *H12*, Fay & Wu's *H*, Fu & Li's *D*, and Fu & Li's *F* statistics of *ACE2* under the X-chromosome-wide background. Each dot denotes the statistical value of *ACE2* estimated within a sliding window of 10 kb in length by an increment of 5 kb for each population. The lines are the X-chromosome-wide distribution estimated in the same way. We grouped continental populations due to their similar profiles along the chromosome,

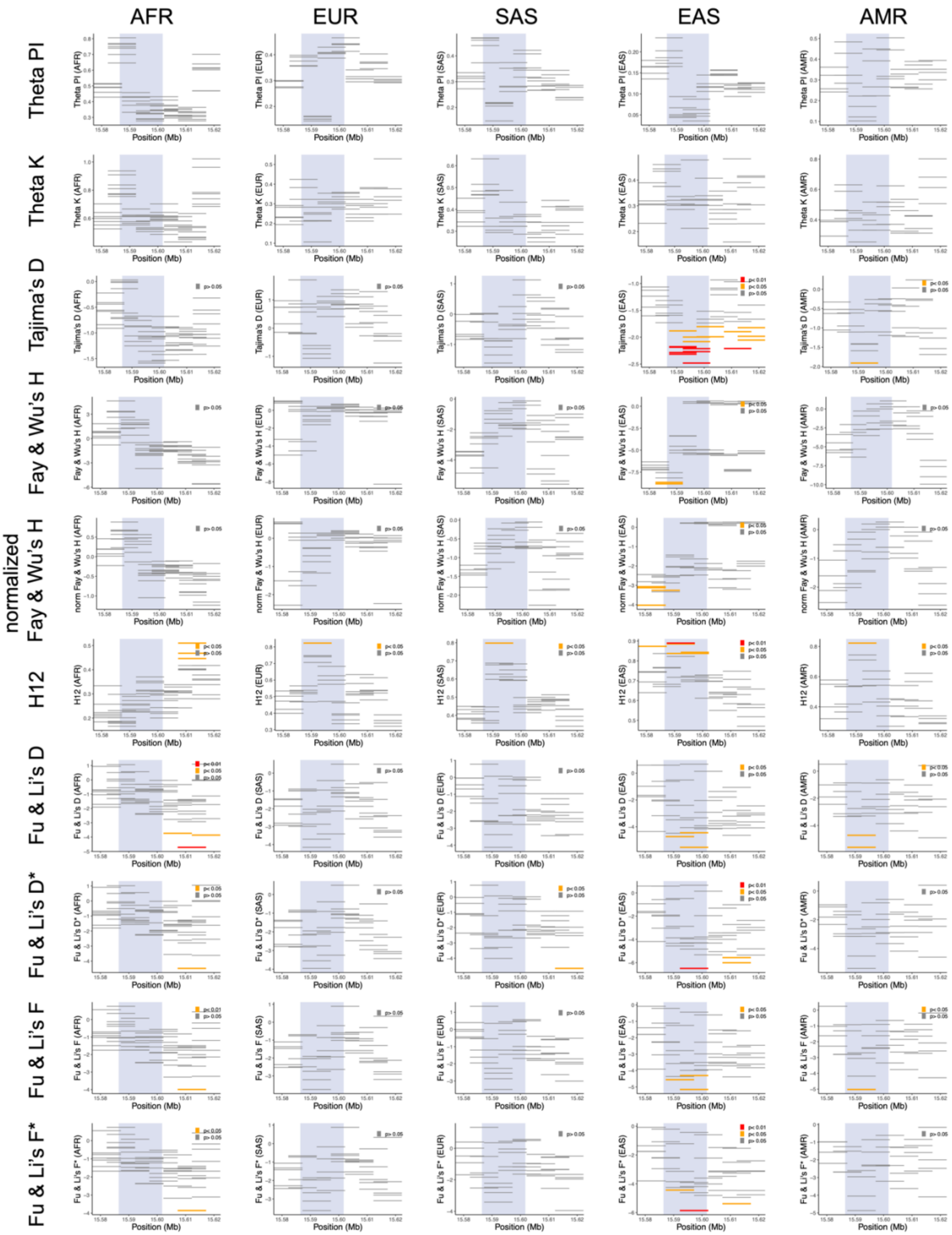


**Figure S13 | Genetic diversity, Tajima's *D*, Fay & Wu's *H*, *H12*, Fu & Li's *D*, and Fu & Li's *F* statistics on *ACE2*.**

Each line represents the estimate of a given statistics within a specific window in one single population. The region (chrX:15,586,447-15,601,720) with shared HIMs between *ACE2*-hg1 and *ACE2*-hg2 were highlighted, covering the most significant signals in EAS.

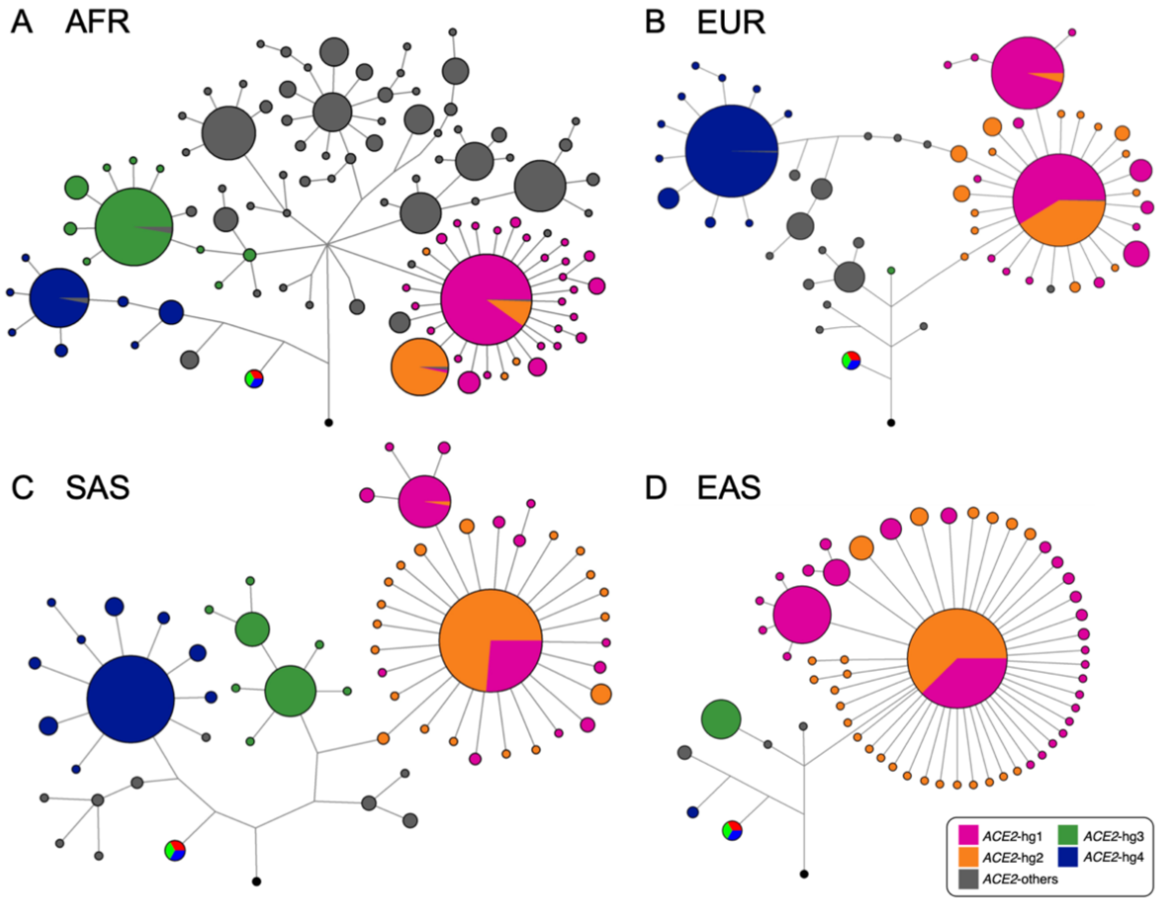


E

| | # branches | # total branches | # sequences | # sequences on the central branch | # total sequences |
|---|---|---|---|---|---|
| AFR | 30 (28.6%) | 105 | 445 (44.4%) | 334 (33.3%) | 1003 |
| EUR | 28 (50.9%) | 55 | 469 (61.2%) | 308 (40.2%) | 766 |
| SAS | 29 (50.0%) | 58 | 501 (69.8%) | 420 (58.5%) | 718 |
| **EAS** | **44 (77.2%)** | **57** | **723 (94.6%)** | **588 (77.0%)** | **764** |

**Figure S14 | Haplotype networks of *ACE2* sequences for the continental groups.**

**A-D.** Haplotype networks of *ACE2* sequences for AFR, EUR, SAS, and EAS populations, respectively; **E.** Numbers of branches and sequences on the nodes of clustering center of *ACE2*-hg1 and *ACE2*-hg2.

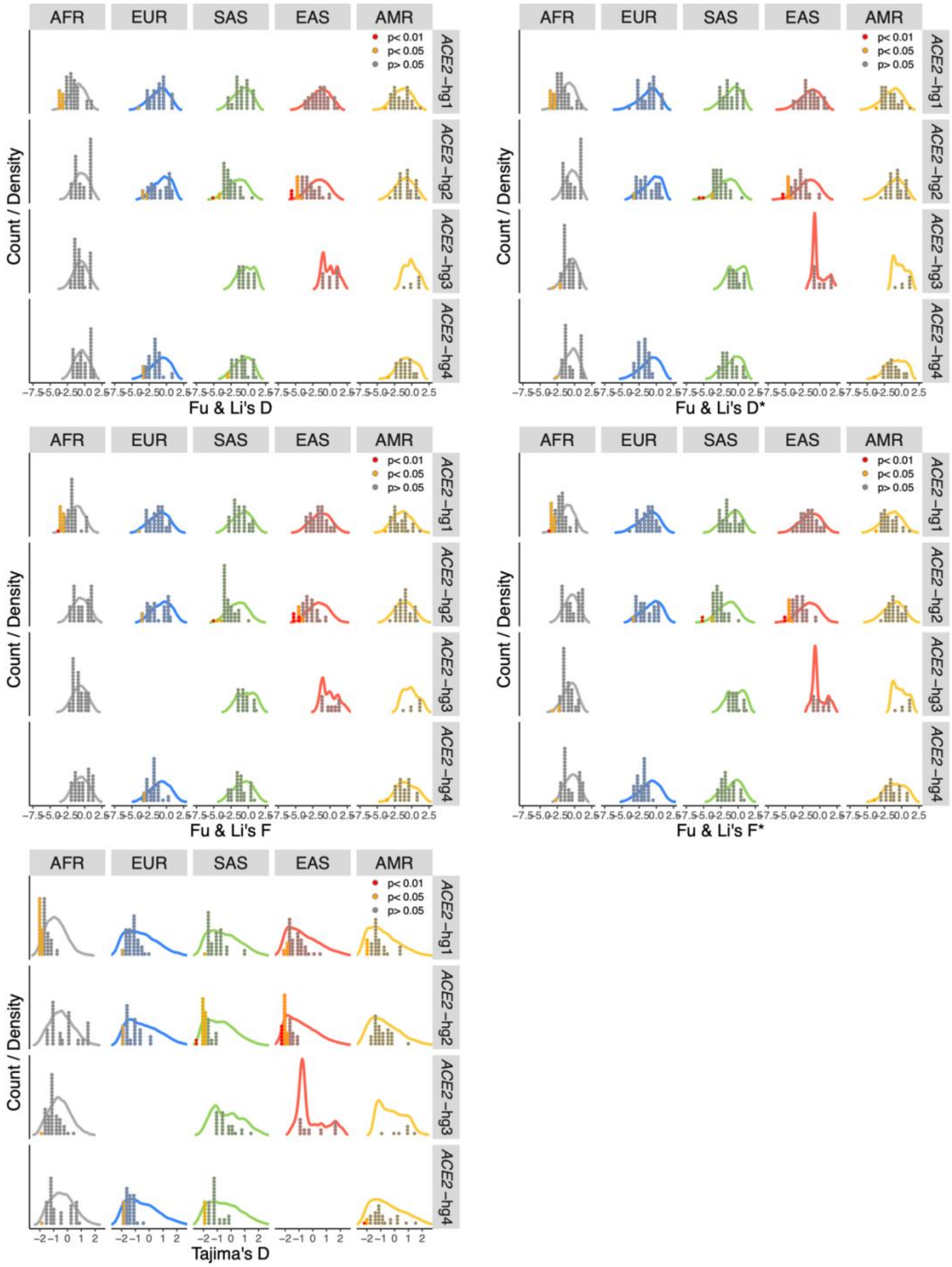


**Figure S15 | Haplogroup-specific Fu & Li's *D*, Fu & Li's *F*, and Tajima's *D* statistics.**

Each dot denotes the statistical values estimated within a sliding window of 10 kb in length by an increment of 5 kb for each population and haplogroup. And the lines are the X-chromosome-wide distribution estimated using the same number of sequences. We grouped continental populations due to their similar profiles.

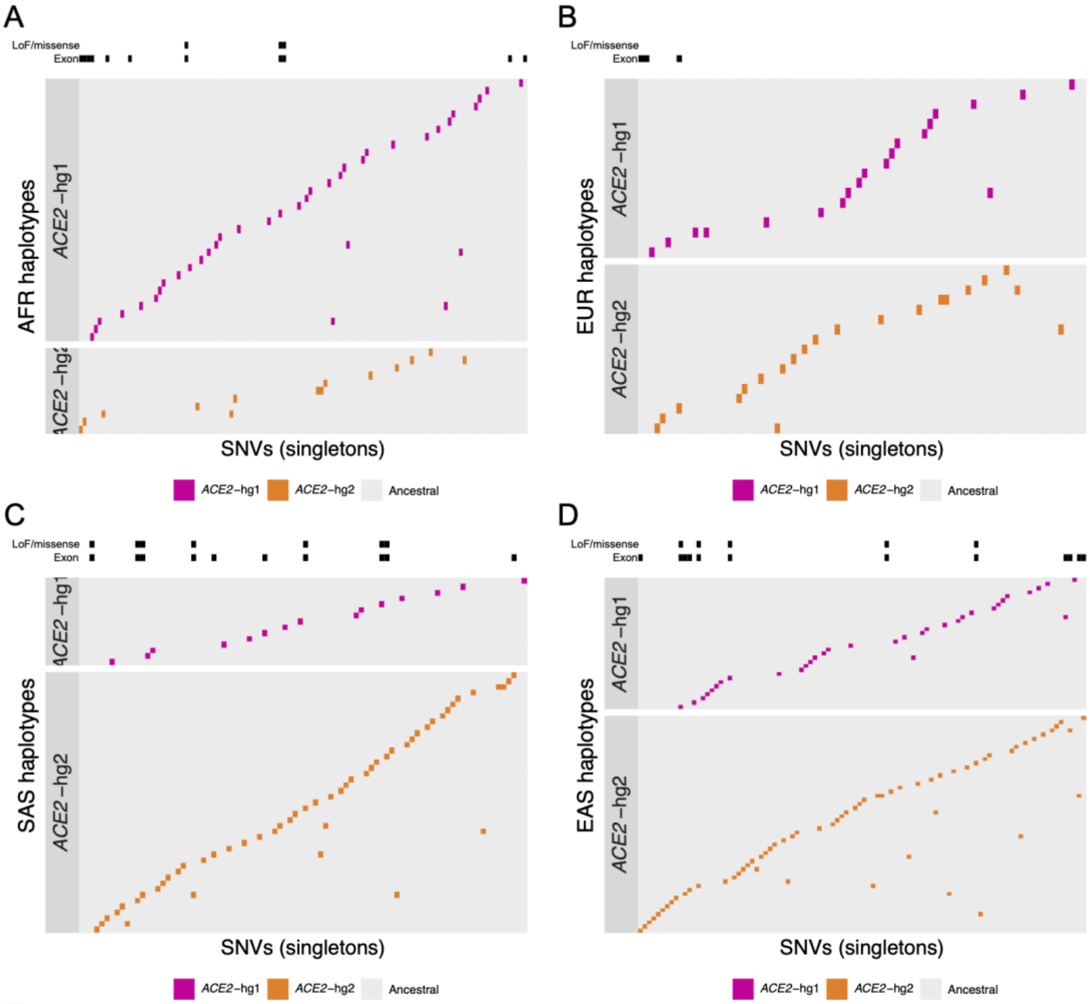


E

| | | # sequences | # singletons | # sequences with singleton | # sequences with >1 singletons |
|---|---|---|---|---|---|
| AFR | All | 1003 | 120 | 99 | 17 |
| | *ACE2*-hg1 | 349 | 39 | 35 | 4 |
| | *ACE2*-hg2 | 93 | 14 | 11 | 3 |
| EUR | All | 766 | 83 | 58 | 8 |
| | *ACE2*-hg1 | 308 | 20 | 18 | 2 |
| | *ACE2*-hg2 | 162 | 21 | 17 | 4 |
| SAS | All | 718 | 89 | 81 | 7 |
| | *ACE2*-hg1 | 170 | 16 | 16 | 0 |
| | *ACE2*-hg2 | 338 | 52 | 45 | 6 |
| EAS | All | 764 | 100 | 87 | 12 |
| | *ACE2*-hg1 | 325 | 34 | 32 | 2 |
| | *ACE2*-hg2 | 408 | 64 | 53 | 10 |

**Figure S16 | Haplotype plot of *ACE2*-hg1 and *ACE2*-hg2 using only singleton variants.**

**A-D.** Haplotype plot of *ACE2* sequences for AFR, EUR, SAS, and EAS populations, respectively. Each column indicates a SNV, and each row represents a sequence. Ancestral and derived alleles were in different colors; **E.** Summary statistics of sequence and singleton counts for *ACE2*-hg1 and *ACE2*-hg2.

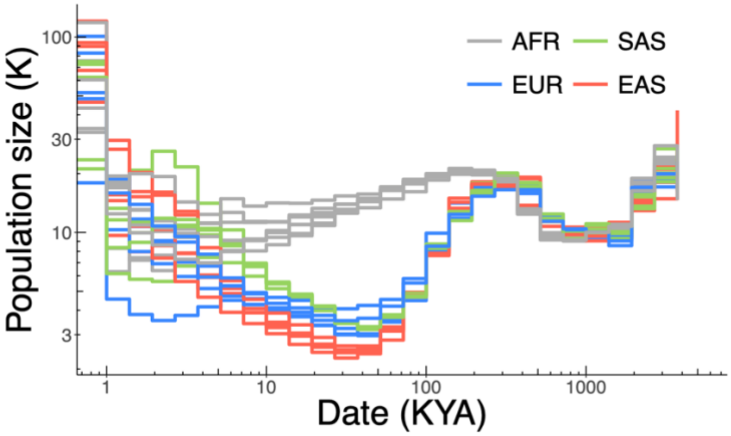


**Figure S17 | Population sizes estimated by Relate using X chromosomes in the 1000 Genomes Project.**

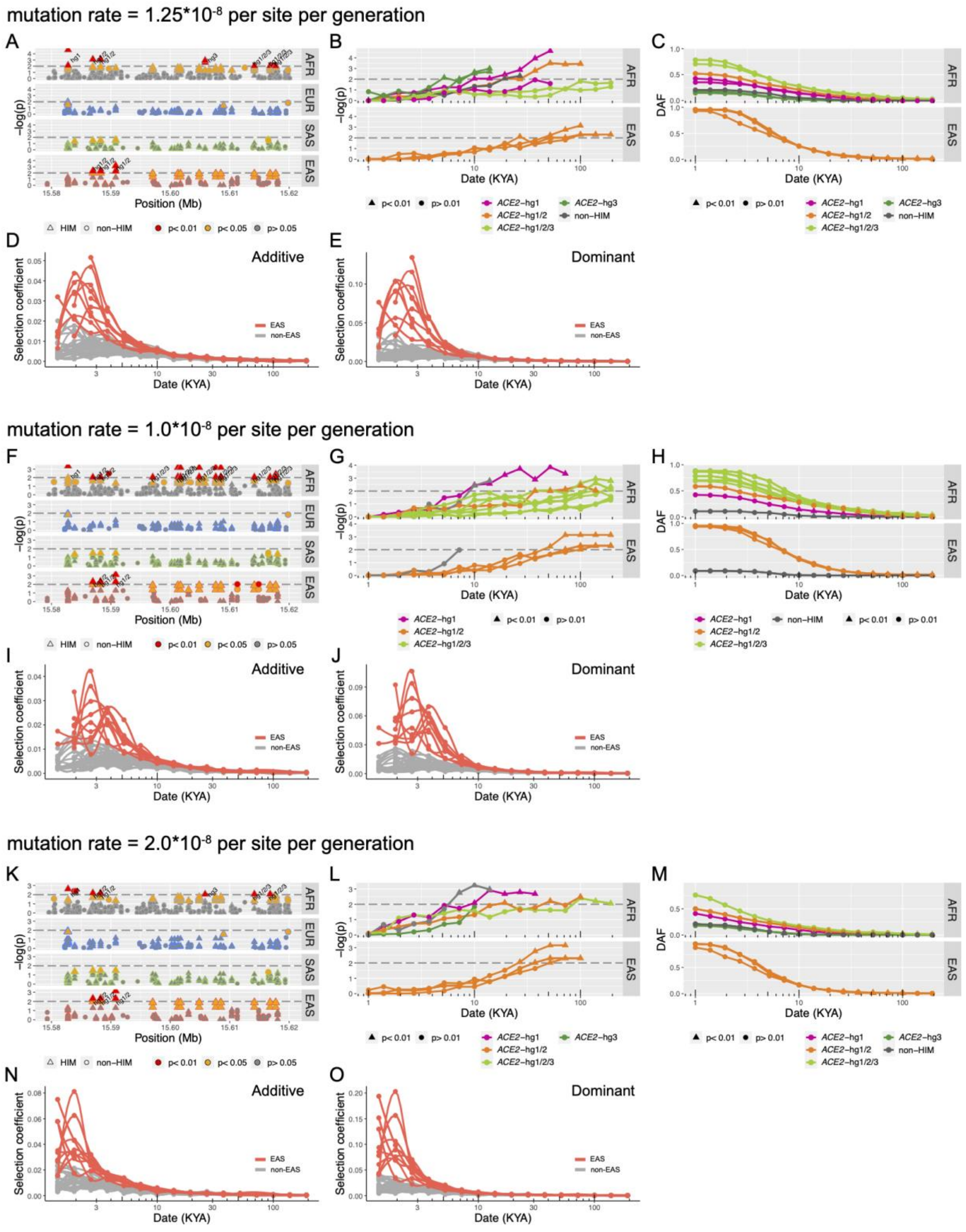


**Figure S18 | Selection evidence of *ACE2* inferred by Relate.**

**A, F, K.** Selection signals at *ACE2* based on different mutation rates; **B, G, L.** Evidence of selection at *ACE2* along with the history; **C, H, M.** Allele frequency change along with the history. Only populations with

selection signals ($P < 0.01$) were presented; **D, I, N.** Theoretical selection coefficients of *ACE2* variants (rs4240157, rs4646174, and rs879922) along with the history. Each curve indicates the changes of selection coefficient of each variant in one single population. The selection coefficient was estimated based on the allele frequency changes inferred by Relate assuming an additive model, without the consideration of genetic drift; **E, J, O.** The selection coefficient assuming a dominant model.

The mutation rate of $1.25 \times 10^{-8}$ per site per generation is widely used as a slow mutation rate for the genome-wide variations on human genome (35). We are also aware that the mutation rate on X chromosome might be lower than that on autosomes, due to more mutations accumulated in male germline compared with the female germline (41), which is known as the “male-driven” evolution (42). We compared the human reference genome with the sequence of chimpanzee (panTro5 from UCSC genome browser). The odds of [the number of different sites between human and chimpanzee on the X chromosome] / [the number of different sites between human and chimpanzee on the autosomes] was about 0.8 in our estimation. We then applied the mutation rate of $1.0 \times 10^{-8}$ per site per generation and a fast one ($2.0 \times 10^{-8}$ per site per generation) in our analysis. The results were similar based on different mutation rates.

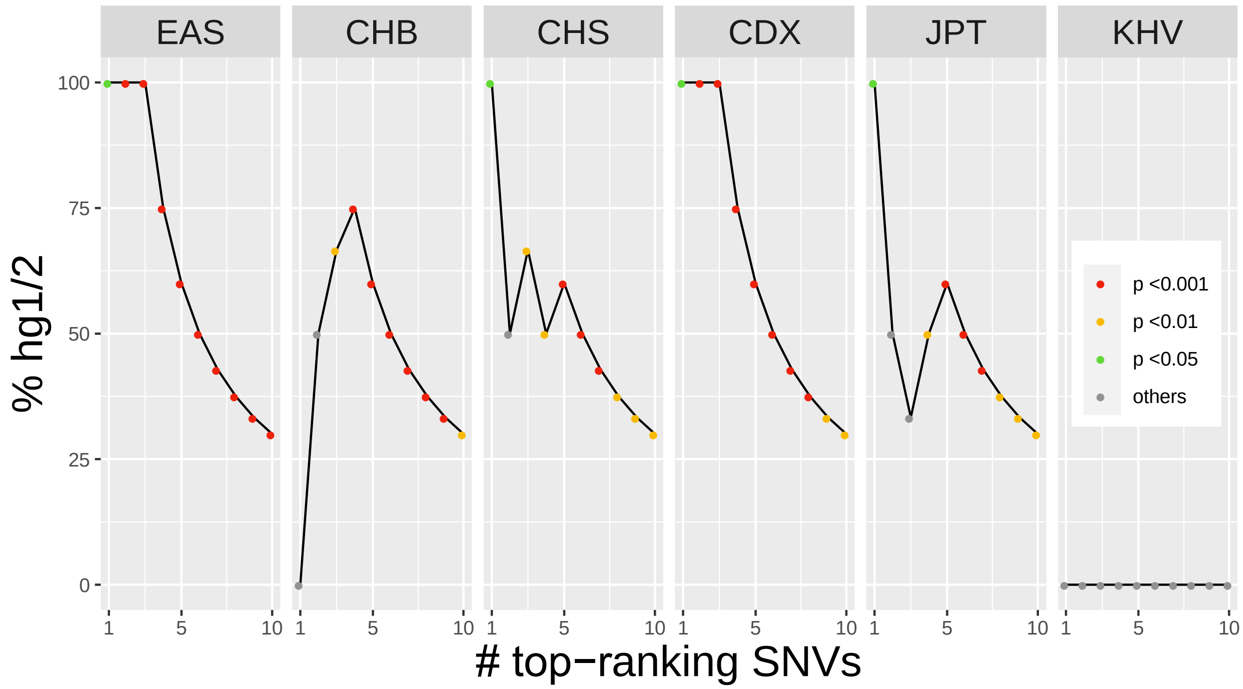


**Figure S19 | Identification of casual variants by iSAFE.**

We performed the analysis of iSAFE within the gene region of *ACE2* on combined EAS populations from KGP dataset and individual EAS populations, respectively. We used the "--SAFE" command due to the short gene length. Variants were ranked in order of their "SAFE" scores. Enrichment analysis was conducted on multiple thresholds of top-ranking variants by fisher exact test. For the analysis based on the combined dataset of EAS populations, all of the 3 HIMs shared by both *ACE2*-hg1 and *ACE2*-hg2 (rs4240157, rs4646174, and rs879922) ranked the top. Exception was observed in KHV, in which population *ACE2*-hg2-specific HIMs ranked the top.

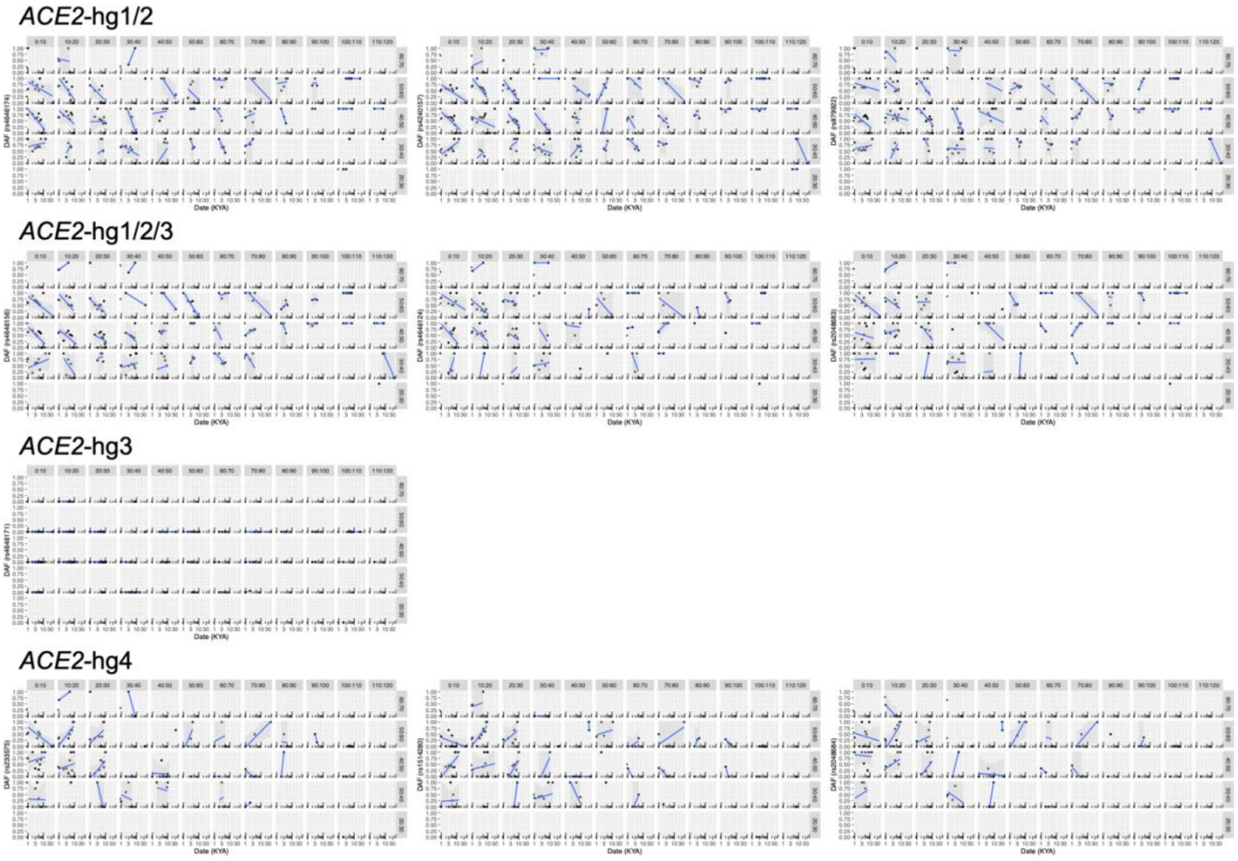


**Figure S20 | Allele frequencies of HIMs in ancient samples across the Eurasian continent.**

Each panel indicates the allele frequencies of one HIM, grouped according to their belonging haplogroups. Ancient samples were grouped according to their coordinate locations and dates. Grids in each panel indicate the regions of given longitude ranges and latitude ranges.

# Supplementary Tables

**Table S1 | Haplogroup informative markers (HIMs) at *ACE2*.**

| SNP ID | POS (b37) | POS (b38) | REF/ALT | ANC/DER | ACE2-hg-1 (ANC/DER) | ACE2-hg-2 (ANC/DER) | ACE2-hg-3 (ANC/DER) | ACE2-hg-4 (ANC/DER) | Pilot mask | Strict mask |
|---|---|---|---|---|---|---|---|---|---|---|
| rs233574 | 15582609 | 15564486 | T/C | C/T | 0 | 0 | 0 | 1 | P | Z |
| rs1514283 | 15582747 | 15564624 | T/C | C/T | 1 | 1 | 0 | 1 | P | P |
| rs1514282 | 15582763 | 15564640 | T/C | C/T | 1 | 1 | 0 | 1 | P | P |
| rs1514281 | 15582786 | 15564663 | T/C | T/C | 0 | 0 | 1 | 0 | P | P |
| rs2074192 | 15582790 | 15564667 | C/T | C/T | 1 | 0 | 0 | 0 | P | P |
| rs233575 | 15582966 | 15564843 | G/A | A/G | 0 | 0 | 0 | 1 | P | P |
| rs714205 | 15583904 | 15565781 | C/G | C/G | 0 | 1 | 0 | 0 | P | P |
| rs1514280 | 15586448 | 15568325 | A/G | G/A | 0 | 0 | 0 | 1 | P | P |
| rs4240157 | 15586964 | 15568841 | C/T | C/T | 1 | 1 | 0 | 0 | P | P |
| rs4646176 | 15587504 | 15569381 | C/G | C/G | 0 | 0 | 1 | 0 | P | P |
| rs4646174 | 15588271 | 15570148 | C/G | C/G | 1 | 1 | 0 | 0 | P | P |
| rs4646171 | 15589028 | 15570905 | T/C | T/C | 0 | 0 | 1 | 0 | P | P |
| rs2301692 | 15590120 | 15571997 | T/C | T/C | 0 | 0 | 1 | 0 | P | P |
| rs2301693 | 15590192 | 15572069 | G/A | G/A | 0 | 0 | 1 | 0 | P | P |
| rs879922 | 15590807 | 15572684 | C/G | C/G | 1 | 1 | 0 | 0 | P | P |
| rs4646156 | 15597043 | 15578920 | A/T | A/T | 1 | 1 | 1 | 0 | P | P |
| rs4646155 | 15597509 | 15579386 | C/T | C/T | 0 | 0 | 1 | 0 | P | P |
| rs4646153 | 15597835 | 15579712 | C/T | T/C | 0 | 0 | 0 | 1 | P | P |
| rs4646152 | 15598024 | 15579901 | A/G | G/A | 0 | 0 | 0 | 1 | P | P |
| rs2048684 | 15600215 | 15582092 | A/C | C/A | 0 | 0 | 0 | 1 | P | P |
| rs2316904 | 15600691 | 15582568 | C/T | T/C | 0 | 0 | 0 | 1 | P | Z |
| rs4646147 | 15600744 | 15582621 | T/A | A/T | 0 | 0 | 0 | 1 | P | Z |
| rs2316903 | 15601274 | 15583151 | G/T | G/T | 1 | 1 | 1 | 0 | P | P |
| rs4646144 | 15601695 | 15583572 | G/A | G/A | 0 | 0 | 1 | 0 | P | Z |
| rs4646143 | 15601719 | 15583596 | T/C | T/C | 1 | 1 | 1 | 0 | P | P |
| rs4646142 | 15603064 | 15584941 | G/C | G/C | 0 | 1 | 0 | 0 | P | P |
| rs1514279 | 15604865 | 15586742 | G/A | G/A | 1 | 1 | 1 | 0 | P | P |
| rs4646140 | 15605852 | 15587729 | C/T | C/T | 0 | 0 | 1 | 0 | P | P |
| rs971249 | 15607650 | 15589527 | T/C | T/C | 1 | 1 | 1 | 0 | P | P |
| rs2158083 | 15608386 | 15590263 | C/T | T/C | 0 | 0 | 0 | 1 | P | P |
| rs2048683 | 15608499 | 15590376 | T/G | T/G | 1 | 1 | 1 | 0 | P | P |
| rs757066 | 15608952 | 15590829 | C/T | T/C | 0 | 0 | 0 | 1 | P | P |
| rs2285666 | 15610348 | 15592225 | C/T | C/T | 0 | 1 | 0 | 0 | P | P |
| rs2023802 | 15614145 | 15596022 | G/A | G/A | 1 | 1 | 1 | 0 | P | P |
| rs4646127 | 15615453 | 15597330 | A/G | G/A | 0 | 0 | 0 | 1 | P | Z |
| rs4646124 | 15616796 | 15598673 | T/C | T/C | 1 | 1 | 1 | 0 | P | P |
| rs4646120 | 15617736 | 15599613 | G/A | G/A | 1 | 1 | 1 | 0 | P | P |
| rs2106809 | 15618061 | 15599938 | A/G | A/G | 0 | 1 | 0 | 0 | P | P |
| rs1978124 | 15618063 | 15599940 | T/C | C/T | 0 | 0 | 0 | 1 | P | P |

"1" denotes the derived allele, and "0" is the ancestral allele. We determined the ancestral and derived alleles according to the ancestral sequences from the 1000 Genome database (e71) (ftp://ftp.1000genomes.ebi.ac.uk/vol1/ftp/phase1/analysis_results/supporting/ance stral_alignments/).

Genome masks were downloaded from the 1000 Genome database (http://ftp.1000genomes.ebi.ac.uk/vol1/ftp/release/20130502/supporting/accessible_genome_masks/ ). "P" denotes the pass of filter and "Z" indicates the lower data quality. For the pilot mask, sites with a depth of coverage of <8960 or >35840 across all samples were excluded; for the strict mask, sites with a depth of coverage of <8960 or >26880 were excluded.

**Table S2 | Haplogroup frequencies of East Asian populations.**

| Pop | #Sequence | Lat. | Long. | *ACE2*-hg1 | *ACE2*-hg2 | *ACE2*-hg3 | *ACE2*-hg4 | *ACE2*-others |
|---|---|---|---|---|---|---|---|---|
| She | 13 | 27.0 | 119.0 | 0.231 | 0.769 | 0.000 | 0.000 | 0.000 |
| Japanese | 35 | 37.5 | 139.0 | 0.371 | 0.629 | 0.000 | 0.000 | 0.000 |
| YaoLB | 59 | 23.7 | 109.2 | 0.322 | 0.610 | 0.034 | 0.000 | 0.034 |
| Lizu | 82 | 20.0 | 110.2 | 0.402 | 0.585 | 0.012 | 0.000 | 0.000 |
| YaoGL | 57 | 25.2 | 110.2 | 0.439 | 0.561 | 0.000 | 0.000 | 0.000 |
| CHS | 158 | 31.2 | 121.5 | 0.418 | 0.557 | 0.025 | 0.000 | 0.000 |
| Han | 62 | 34.7 | 107.8 | 0.387 | 0.548 | 0.048 | 0.000 | 0.016 |
| JPT | 152 | 35.7 | 139.7 | 0.428 | 0.546 | 0.026 | 0.000 | 0.000 |
| Dai | 11 | 21.0 | 100.0 | 0.455 | 0.545 | 0.000 | 0.000 | 0.000 |
| Daur | 11 | 48.5 | 124.0 | 0.455 | 0.545 | 0.000 | 0.000 | 0.000 |
| CDX | 142 | 22.0 | 100.8 | 0.380 | 0.542 | 0.049 | 0.007 | 0.021 |
| Tu | 13 | 36.0 | 101.0 | 0.462 | 0.538 | 0.000 | 0.000 | 0.000 |
| Hezhen | 13 | 47.5 | 133.5 | 0.385 | 0.538 | 0.077 | 0.000 | 0.000 |
| Balti | 48 | 35.4 | 75.9 | 0.313 | 0.521 | 0.000 | 0.125 | 0.042 |
| KHV | 152 | 10.8 | 106.6 | 0.447 | 0.520 | 0.026 | 0.000 | 0.007 |
| Deng | 67 | 29.7 | 94.4 | 0.493 | 0.507 | 0.000 | 0.000 | 0.000 |
| CHB | 160 | 39.9 | 116.4 | 0.450 | 0.506 | 0.031 | 0.006 | 0.006 |
| Naxi | 10 | 26.0 | 100.0 | 0.400 | 0.500 | 0.000 | 0.000 | 0.100 |
| Mongola | 12 | 48.5 | 119.0 | 0.500 | 0.500 | 0.000 | 0.000 | 0.000 |
| Oroqen | 12 | 50.4 | 126.5 | 0.417 | 0.500 | 0.083 | 0.000 | 0.000 |
| Hui | 352 | 38.0 | 102.5 | 0.463 | 0.494 | 0.026 | 0.009 | 0.009 |
| Dolan | 281 | 40.1 | 80.7 | 0.356 | 0.459 | 0.025 | 0.128 | 0.032 |
| Tibetan | 48 | 29.7 | 91.2 | 0.479 | 0.458 | 0.042 | 0.000 | 0.021 |
| Gongbo | 22 | 29.7 | 94.4 | 0.409 | 0.455 | 0.091 | 0.000 | 0.045 |
| Yakut | 32 | 63.0 | 129.5 | 0.406 | 0.438 | 0.094 | 0.031 | 0.031 |
| Uygur | 149 | 44.0 | 81.0 | 0.396 | 0.430 | 0.000 | 0.148 | 0.027 |
| Tujia | 10 | 29.0 | 109.0 | 0.600 | 0.400 | 0.000 | 0.000 | 0.000 |
| Miaozu | 13 | 28.0 | 109.0 | 0.462 | 0.385 | 0.154 | 0.000 | 0.000 |
| LopNur | 263 | 38.8 | 82.9 | 0.354 | 0.376 | 0.179 | 0.084 | 0.008 |
| Luoba | 61 | 29.7 | 94.4 | 0.541 | 0.361 | 0.098 | 0.000 | 0.000 |
| Keriya | 101 | 39.0 | 84.2 | 0.396 | 0.356 | 0.020 | 0.099 | 0.129 |
| Lahu | 9 | 22.0 | 100.0 | 0.667 | 0.333 | 0.000 | 0.000 | 0.000 |
| Kazak | 42 | 48.0 | 87.0 | 0.429 | 0.310 | 0.024 | 0.214 | 0.024 |
| Cambodians | 13 | 12.0 | 105.0 | 0.692 | 0.308 | 0.000 | 0.000 | 0.000 |
| Tajik | 43 | 37.5 | 71.6 | 0.465 | 0.256 | 0.047 | 0.233 | 0.000 |
| Xibo | 10 | 43.5 | 81.5 | 0.800 | 0.200 | 0.000 | 0.000 | 0.000 |
| Yizu | 11 | 28.0 | 103.0 | 0.818 | 0.182 | 0.000 | 0.000 | 0.000 |

The table was presented by sorting the “*ACE2*-hg2” column.

**Table S3 | Characteristics of the COVID-19 patients in this study.**

| | | Asymptomatic | Mild | Moderate | Severe | Critical | Mild (broad) | Severe (broad) | Total |
|---|---|---|---|---|---|---|---|---|---|
| Gender | Female | 64 | 16 | 310 | 169 | 69 | 390 | 238 | 628 |
| | Male | 50 | 17 | 254 | 180 | 100 | 321 | 280 | 601 |
| Age (Female) | 10-30 | 12 | 3 | 34 | 0 | 2 | 49 | 2 | 51 |
| | 30-50 | 29 | 8 | 93 | 19 | 17 | 130 | 36 | 166 |
| | 50-70 | 21 | 4 | 150 | 97 | 33 | 175 | 130 | 305 |
| | >70 | 2 | 1 | 33 | 53 | 17 | 36 | 70 | 106 |
| Age (Male) | 10-30 | 10 | 5 | 29 | 0 | 2 | 44 | 2 | 46 |
| | 30-50 | 18 | 5 | 90 | 38 | 23 | 113 | 61 | 174 |
| | 50-70 | 20 | 5 | 102 | 94 | 51 | 127 | 145 | 272 |
| | >70 | 2 | 2 | 33 | 48 | 24 | 37 | 72 | 109 |
| *ACE2*-hg (Female) | hg1/1 | 6 | 4 | 57 | 33 | 13 | 67 | 46 | 113 |
| | hg1/2 | 31 | 8 | 147 | 66 | 29 | 186 | 95 | 281 |
| | hg1/3 | 2 | 1 | 4 | 3 | 1 | 7 | 4 | 11 |
| | hg1/4 | 1 | 0 | 0 | 1 | 0 | 1 | 1 | 2 |
| | hg1/others | 1 | 0 | 0 | 0 | 0 | 1 | 0 | 1 |
| | hg2/2 | 19 | 2 | 93 | 58 | 23 | 114 | 81 | 195 |
| | hg2/3 | 3 | 1 | 5 | 8 | 2 | 9 | 10 | 19 |
| | hg2/4 | 0 | 0 | 3 | 0 | 1 | 3 | 1 | 4 |
| | hg2/others | 1 | 0 | 1 | 0 | 0 | 2 | 0 | 2 |
| *ACE2*-hg (Male) | hg1 | 15 | 3 | 103 | 89 | 49 | 121 | 138 | 259 |
| | hg2 | 35 | 12 | 147 | 88 | 51 | 194 | 139 | 333 |
| | hg3 | 0 | 2 | 3 | 3 | 0 | 5 | 3 | 8 |
| | hg4 | 0 | 0 | 0 | 0 | 0 | 0 | 0 | 0 |
| | others | 0 | 0 | 1 | 0 | 0 | 1 | 0 | 1 |

**Table S4 | Summary statistics of variants associated with COVID-19 susceptibility from the COVID19-hg database.**

| rsID | *ACE2*-hg | REF | ANC | $\beta$ | *P* | # samples | AF | file |
|---|---|---|---|---|---|---|---|---|
| rs2074192 | hg1 | C | C | 0.038 | 0.007 | 938473 | 0.5653 | COVID19_HGI_B2_ALL_leave_23andme_20201020 |
| rs2074192 | hg1 | C | C | 0.026 | 0.028 | 1248525 | 0.4756 | COVID19_HGI_C2_ALL_leave_23andme_20201020 |
| rs2074192 | hg1 | C | C | 0.046 | 0.049 | 612774 | 0.6139 | COVID19_HGI_A2_ALL_leave_23andme_20201020 |
| rs714205 | hg2 | C | C | -0.063 | 0.034 | 612774 | 0.2154 | COVID19_HGI_A2_ALL_leave_23andme_20201020 |
| rs1514280 | hg4 | A | G | 0.036 | 0.019 | 939689 | 0.7999 | COVID19_HGI_B2_ALL_leave_23andme_20201020 |
| rs1514280 | hg4 | A | G | 0.026 | 0.044 | 1249702 | 0.6763 | COVID19_HGI_C2_ALL_leave_23andme_20201020 |
| rs1514280 | hg4 | A | G | 0.034 | 0.025 | 903672 | 0.8001 | COVID19_HGI_B2_ALL_eur_leave_23andme_20201020 |
| rs4646174 | hg1/2 | C | C | 0.031 | 0.043 | 903672 | 0.7979 | COVID19_HGI_B2_ALL_eur_leave_23andme_20201020 |
| rs4646174 | hg1/2 | C | C | 0.028 | 0.039 | 941960 | 0.7931 | COVID19_HGI_B2_ALL_leave_23andme_20201020 |
| rs4646142 | hg2 | G | G | -0.071 | 0.013 | 612774 | 0.2507 | COVID19_HGI_A2_ALL_leave_23andme_20201020 |
| rs2285666 | hg2 | C | C | -0.074 | 0.010 | 612774 | 0.2469 | COVID19_HGI_A2_ALL_leave_23andme_20201020 |

**Table S5 | COVID19Genes on the X chromosome**

| Ensemble ID | Start position | End position | Symbol | Gene type | Reference |
|---|---|---|---|---|---|
| ENSG00000102010 | 15482369 | 15574652 | *BMX* | protein_coding | (43) |
| ENSG00000130234 | 15579156 | 15620271 | *ACE2* | protein_coding | (44-47) |
| ENSG00000147003 | 15645441 | 15683154 | *TMEM27* | protein_coding | (43) |
| ENSG00000186312 | 15693055 | 15721847 | *CA5BP1* | pseudogene | (43) |
| ENSG00000169239 | 15706953 | 15805747 | *CA5B* | protein_coding | (43) |
| ENSG00000101868 | 24712036 | 25015103 | *POLA1* | protein_coding | (48) |
| ENSG00000068400 | 48830134 | 48858675 | *GRIPAP1* | protein_coding | (48) |
| ENSG00000120509 | 69506445 | 69510364 | *PDZD11* | protein_coding | (48) |
| ENSG00000147140 | 70503042 | 70521018 | *NONO* | protein_coding | (49) |
| ENSG00000101811 | 100075384 | 100095921 | *CSTF2* | protein_coding | (49) |
| ENSG00000102393 | 100652791 | 100662913 | *GLA* | protein_coding | (48) |
| ENSG00000089682 | 106307650 | 106362057 | *RBM41* | protein_coding | (48) |
| ENSG00000102024 | 114795501 | 114885181 | *PLS3* | protein_coding | (50) |
| ENSG00000171004 | 131760044 | 132095423 | *HS6ST2* | protein_coding | (48) |
| ENSG00000071553 | 153656978 | 153664862 | *ATP6AP1* | protein_coding | (48) |

## References


1. Danecek, P, Auton, A, Abecasis, G, *et al.* The variant call format and VCFtools. *Bioinformatics*. 2011; **27**(15): 2156-8.
2. Hinch, AG, Tandon, A, Patterson, N, *et al.* The landscape of recombination in African Americans. *Nature*. 2011; **476**(7359): 170-U67.
3. McVean, G, Awadalla, P, Fearnhead, P. A coalescent-based method for detecting and estimating recombination from gene sequences. *Genetics*. 2002; **160**(3): 1231-41.
4. Kong, A, Thorleifsson, G, Gudbjartsson, DF, *et al.* Fine-scale recombination rate differences between sexes, populations and individuals. *Nature*. 2010; **467**(7319): 1099-103.
5. Peckham, H, de Gruijter, NM, Raine, C, *et al.* Male sex identified by global COVID-19 meta-analysis as a risk factor for death and ITU admission. *Nature communications*. 2020; **11**(1): 1-10.
6. Altshuler, DM, Durbin, RM, Abecasis, GR, *et al.* A global reference for human genetic variation. *Nature*. 2015; **526**(7571): 68-+.
7. Pagani, L, Lawson, DJ, Jagoda, E, *et al.* Genomic analyses inform on migration events during the peopling of Eurasia. *Nature*. 2016; **538**(7624): 238-+.
8. Bergström, A, McCarthy, SA, Hui, R, *et al.* Insights into human genetic variation and population history from 929 diverse genomes. *Science*. 2020; **367**(6484).
9. Mallick, S, Li, H, Lipson, M, *et al.* The Simons genome diversity project: 300 genomes from 142 diverse populations. *Nature*. 2016; **538**(7624): 201-6.
10. Mondal, M, Casals, F, Xu, T, *et al.* Genomic analysis of Andamanese provides insights into ancient human migration into Asia and adaptation. *Nature Genetics*. 2016; **48**(9): 1066-70.
11. Zhang, C, Gao, Y, Ning, Z, *et al.* PGG.SNV: understanding the evolutionary and medical implications of human single nucleotide variations in diverse populations. *Genome biology*. 2019; **20**(1): 215.
12. Wang, F, Huang, S, Gao, R, *et al.* Initial whole-genome sequencing and analysis of the host genetic contribution to COVID-19 severity and susceptibility. *Cell discovery*. 2020; **6**(1): 1-16.
13. Wu, P, Chen, D, Ding, W, *et al.* The trans-omics landscape of COVID-19. *Nature Communications*. 2021; **12**(1): 4543.
14. Zhu, H, Zheng, F, Li, L, *et al.* A Chinese host genetic study discovered type I interferons and causality of cholesterol levels and WBC counts on COVID-19 severity. 2021.
15. Mathieson, I, Lazaridis, I, Rohland, N, *et al.* Genome-wide patterns of selection in 230 ancient Eurasians. *Nature*. 2015; **528**(7583): 499-+.
16. Prufer, K, Racimo, F, Patterson, N, *et al.* The complete genome sequence of a Neanderthal from the Altai Mountains. *Nature*. 2014; **505**(7481): 43-+.
17. Prufer, K, de Filippo, C, Grote, S, *et al.* A high-coverage Neandertal genome from Vindija Cave in Croatia. *Science*. 2017; **358**(6363): 655-8.
18. Meyer, M, Kircher, M, Gansauge, MT, *et al.* A High-Coverage Genome Sequence from an Archaic Denisovan Individual. *Science*. 2012; **338**(6104): 222-6.
19. Initiative, C-HG. The COVID-19 Host Genetics Initiative, a global initiative to elucidate the role of host genetic factors in susceptibility and severity of the SARS-CoV-2 virus pandemic. *European Journal of Human Genetics*. 2020; **28**(6): 715.
20. Delaneau, O, Marchini, J, Zagury, JF. A linear complexity phasing method for thousands of genomes. *Nat Methods*. 2012; **9**(2): 179-81.
21. Howe, KL, Achuthan, P, Allen, J, *et al.* Ensembl 2021. *Nucleic Acids Research*. 2021; **49**(D1): D884-D91.
22. Tajima, F. Statistical-method for testing the neutral mutation hypothesis by DNA polymorphism. *Genetics*. 1989; **123**(3): 585-95.
23. Fay, JC, Wu, CI. Hitchhiking under positive Darwinian selection. *Genetics*. 2000; **155**(3): 1405-13.
24. Zeng, K, Fu, YX, Shi, SH, *et al.* Statistical tests for detecting positive selection by utilizing high-frequency variants. *Genetics*. 2006; **174**(3): 1431-9.
25. Garud, NR, Messer, PW, Buzbas, EO, *et al.* Recent Selective Sweeps in North American Drosophila melanogaster Show Signatures of Soft Sweeps. *PLoS Genet*. 2015; **11**(2): 32.
26. Harris, AM, DeGiorgio, M. Identifying and Classifying Shared Selective Sweeps from Multilocus Data. *Genetics*. 2020; **215**(1): 143-71.
27. Harris, AM, Garud, NR, DeGiorgio, M. Detection and Classification of Hard and Soft Sweeps from Unphased Genotypes by Multilocus Genotype Identity. *Genetics*. 2018; **210**(4): 1429-52.
28. Fu, YX, Li, WH. STATISTICAL TESTS OF NEUTRALITY OF MUTATIONS. *Genetics*. 1993; **133**(3): 693-709.

29. Souilmi, Y, Lauterbur, ME, Tobler, R, *et al.* An ancient viral epidemic involving host coronavirus interacting genes more than 20,000 years ago in East Asia. *Current biology : CB*. 2021.
30. Zeng, K, Shi, S, Wut, CI. Compound tests for the detection of hitchhiking under positive selection. *Molecular Biology and Evolution*. 2007; **24**(8): 1898-908.
31. Yu, GC, Smith, DK, Zhu, HC, *et al.* GGTREE: an R package for visualization and annotation of phylogenetic trees with their covariates and other associated data. *Methods Ecol Evol*. 2017; **8**(1): 28-36.
32. Bandelt, HJ, Forster, P, Rohl, A. Median-joining networks for inferring intraspecific phylogenies. *Mol Biol Evol*. 1999; **16**(1): 37-48.
33. Nei, M, Li, WH. Mathematical-model for studying genetic-variation in terms of restriction endonucleases. *Proc Natl Acad Sci U S A*. 1979; **76**(10): 5269-73.
34. Lu, DS, Lou, HY, Yuan, K, *et al.* Ancestral Origins and Genetic History of Tibetan Highlanders. *Am J Hum Genet*. 2016; **99**(3): 580-94.
35. Speidel, L, Forest, M, Shi, S, *et al.* A method for genome-wide genealogy estimation for thousands of samples. *Nature genetics*. 2019; **51**(9): 1321-9.
36. Roach, JC, Glusman, G, Smit, AFA, *et al.* Analysis of Genetic Inheritance in a Family Quartet by Whole-Genome Sequencing. *Science*. 2010; **328**(5978): 636-9.
37. Kong, A, Frigge, ML, Masson, G, *et al.* Rate of de novo mutations and the importance of father's age to disease risk. *Nature*. 2012; **488**(7412): 471-5.
38. Conrad, DF, Keebler, JEM, DePristo, MA, *et al.* Variation in genome-wide mutation rates within and between human families. *Nature Genetics*. 2011; **43**(7): 712-U137.
39. Kanehisa, M, Furumichi, M, Tanabe, M, *et al.* KEGG: new perspectives on genomes, pathways, diseases and drugs. *Nucleic Acids Research*. 2017; **45**(D1): D353-D61.
40. Barrett, JC, Fry, B, Maller, J, *et al.* Haploview: analysis and visualization of LD and haplotype maps. *Bioinformatics*. 2005; **21**(2): 263-5.
41. Haldane, J. The mutation rate of the gene for haemophilia, and its segregation ratios in males and females. *Annals of Human Genetics*. 1946; **13**(1): 262-71.
42. Miyata, T, Hayashida, H, Kuma, K, *et al.* Male-driven molecular evolution: a model and nucleotide sequence analysis. In: *Cold Spring Harbor symposia on quantitative biology, 1987*, p. 863-7. Cold Spring Harbor Laboratory Press.
43. Cao, Y, Li, L, Feng, Z, *et al.* Comparative genetic analysis of the novel coronavirus (2019-nCoV/SARS-CoV-2) receptor ACE2 in different populations. *Cell discovery*. 2020; **6**(1): 1-4.
44. Li, W, Moore, MJ, Vasilieva, N, *et al.* Angiotensin-converting enzyme 2 is a functional receptor for the SARS coronavirus. *Nature*. 2003; **426**(6965): 450-4.
45. Wang, Q, Zhang, Y, Wu, L, *et al.* Structural and functional basis of SARS-CoV-2 entry by using human ACE2. *Cell*. 2020; **181**(4): 894-904. e9.
46. Yan, R, Zhang, Y, Li, Y, *et al.* Structural basis for the recognition of SARS-CoV-2 by full-length human ACE2. *Science*. 2020; **367**(6485): 1444-8.
47. Letko, M, Marzi, A, Munster, V. Functional assessment of cell entry and receptor usage for SARS-CoV-2 and other lineage B betacoronaviruses. *Nat Microbiol*. 2020.
48. Gordon, DE, Jang, GM, Bouhaddou, M, *et al.* A SARS-CoV-2 protein interaction map reveals targets for drug repurposing. *Nature*. 2020; **583**(7816): 459-68.
49. Sun, L, Li, P, Ju, X, *et al.* In vivo structural characterization of the whole SARS-CoV-2 RNA genome identifies host cell target proteins vulnerable to re-purposed drugs. *Biorxiv*. 2020.
50. Taylor, K, Das, S, Pearson, M, *et al.* Analysis of genetic host response risk factors in severe COVID-19 patients. *medRxiv*. 2020.
